# Development of a Live Recombinant BCG Expressing Human Immunodeficiency Virus Type 1 (HIV-1) Gag Using a pMyong2 Vector System: Potential Use As a Novel HIV-1 Vaccine

**DOI:** 10.3389/fimmu.2018.00643

**Published:** 2018-03-27

**Authors:** Byoung-Jun Kim, Bo-Ram Kim, Yoon-Hoh Kook, Bum-Joon Kim

**Affiliations:** ^1^Department of Microbiology and Immunology, Biomedical Sciences, College of Medicine, Liver Research Institute, Seoul National University, Seoul, South Korea; ^2^Department of Microbiology and Immunology, Biomedical Sciences, College of Medicine, Cancer Research Institute, Seoul National University, Seoul, South Korea

**Keywords:** *Mycobacterium*, pMyong2 vector system, recombinant *Mycobacterium bovis* BCG, human immunodeficiency virus type 1 Gag p24, human immunodeficiency virus type 1 vaccine

## Abstract

Even though the rate of new human immunodeficiency virus type 1 (HIV-1) infections is gradually decreasing worldwide, an effective preventive vaccine for HIV-1 is still urgently needed. The recombinant *Mycobacterium bovis* BCG (rBCG) is promising for the development of an HIV-1 vaccine. Recently, we showed that a recombinant *Mycobacterium smegmatis* expressing HIV-1 gag in a pMyong2 vector system (rSmeg-pMyong2-p24) increased the efficacy of a vaccine against HIV-1 in mice. Here, we evaluated the potential of an rBCG expressing HIV-1 p24 antigen Gag in pMyong2 (rBCG-pMyong2-p24) in a vaccine application for HIV-1 infection. We found that rBCG-pMyong2-p24 elicited an enhanced HIV-1 p24 Gag expression in rBCG and infected antigen-presenting cells. We also found that compared to rBCG-pAL-p24 in a pAL5000 derived vector system, rBCG-pMyong2-p24 elicited enhanced p24-specific immune responses in vaccinated mice as evidenced by higher levels of HIV-1 Gag-specific CD4 and CD8 T lymphocyte proliferation, gamma interferon ELISPOT cell induction, antibody production, and cytotoxic T lymphocytes (CTL) responses. Furthermore, rBCG-pMyong2-p24 showed a higher level of p24-specific Ab production than rSmeg-pMyong2-p24 in the same pMyong2 vector system. In conclusion, our data indicated that a live recombinant BCG expressing HIV-1 Gag using a pMyong2 vector system, rBCG-pMyong2-p24 elicited an enhanced immune response against HIV-1 infections in a mouse model system. So, rBCG-pMyong2-p24 may have the potential as a prime vaccine in a heterologous prime-boost vaccine strategy for HIV-1 infection.

## Introduction

Despite the contribution of highly activated antiretroviral therapy in controlling human immunodeficiency virus (HIV) replication in infected individuals (1, 2), several problems, including the emergence of drug-resistant viruses after long-term treatment and expensive drug costs, remain to be resolved. Thus, although the rate of new human immunodeficiency virus type 1 (HIV-1) infections is gradually decreasing worldwide, an effective preventive vaccine is still urgently needed to inhibit the further spread of the virus (3, 4). Since an effective immune response against HIV-1 can arise, characterized by HIV-specific T cells with poly-functionality and capacity to proliferate against both the immunodominant viral peptides (5–7), cellular immunity, particularly virus-specific cytotoxic T lymphocytes (CTL), should be a more important component of the host immune system for protection against HIV-1. Based on these findings, several strategies, including the use of live viral vectors and plasmid DNA vaccines, are in development to elicit strong HIV-1-specific CTL and Th1 type response. However, several problems are associated with each of these approaches, including safety issues, which could limit their practical use (8–10).

*Mycobacterium bovis* BCG (BCG), which is currently the most widely administered vaccine worldwide, is the only live attenuated vaccine used to protect against tuberculosis (TB) and has been used for more than 80 years (11–14). Since BCG can prevent disseminated disease in children, it has been used as a part of the World Health Organization Expanded Program on Immunization for childhood vaccination since the early 1970s (15, 16).

The recombinant form of BCG, i.e., recombinant *Mycobacterium bovis* BCG (rBCG), which has been successfully used to express foreign antigens and induce immune responses, has been considered a vaccine candidate against various infectious agents, including *Borrelia burgdorferi, Streptococcus pneumoniae, Bordetella pertussis*, rodent malaria, *Leishmania*, measles virus (17–22), HIV-1, and simian immunodeficiency virus (23, 24). The most practical advantage of the rBCG vector is its high safety. In addition, rBCG demonstrates excellent adjuvant properties, induces long-lasting cellular immune responses that are maintained for at least 1–2 years, has a low production cost, is easy to administer, and usually requires only one or two immunizations (25–27). Therefore, the above-mentioned advantages of the rBCG-based vaccine over other recombinant vaccine approaches suggest that rBCG could be a potent vaccine against HIV-1 infection capable of inducing safe, virus-specific immune responses.

Despite the promise of the rBCG vector as a potential HIV-1 vaccine, its practical applications as an HIV-1 vaccine is limited because of the low immunogenicity due to the lack of stability and amounts in the heterologous expression of foreign genes within rBCG (8, 28, 29). Therefore, to obtain sufficient immunogenicity and elicit protective vaccine efficacy, high rBCG doses of approximately 10- to 100-fold of that needed for a practical BCG vaccination against TB in humans (23) are needed. The *in vivo* administration of high doses of BCG may increase the risk of disseminated BCG in immuno-compromised vaccines or act as a driving force for the replication and dissemination of HIV-1 by hyperactivating T cells (30, 31). We have recently identified a novel human pathogenic member of the *M. avium* complex, *Mycobacterium yongonense* (32). By conducting a genome analysis of *M. yongonense* DSM 45126^T^ (33–37), we also introduced a novel *Mycobacterium–Escherichia coli* shuttle vector system using the mycobacterial replicon of pMyong2, which is a linear plasmid within its genome that can lead to increased heterologous gene expression in recombinant *M. smegmatis* (rSmeg) and rBCG compared to that using the conventional pAL5000 vector system which was originated from *M. fortuitum* (38, 39). Furthermore, we showed that rSmeg expressing HIV-1 p24 Gag using a pMyong2 vector system led to the enhanced immune responses against HIV-1 p24 Gag in mice, compared to rSmeg in the pAL5000 vector system or using an integrative plasmid, pMV306 system, suggesting the feasibility of the pMyong2 vector system in rSmeg vaccine application (40).

Because low-dose immunization of rBCG has been recommended to ensure safety in prophylactic vaccination against HIV-1 (41, 42), in this study, we sought to develop an rBCG vaccine for HIV-1 protection that could be efficacious even in lower doses required for human vaccination. HIV-1 Gag-specific CD8+ T cell responses may be critical in the immune control of HIV infection. So, we have chosen the HIV-1 Gag p24 as the target antigen to express in the pMyong2 vector system (43–45). Therefore, we adopted a pMyong2 vector system to enhance the expression of the foreign HIV-1 p24 Gag gene within rBCG (rBCG-pMyong2-p24). The potency of the pMyong2 vector system, i.e., its enhanced recombinant protein production, was demonstrated in rBCG and the primary bone marrow-derived dendritic cells (BMDCs) infected with rBCG. In addition, to demonstrate its vaccine efficacy, we explored its cellular and humoral immune responses against HIV Gag proteins in vaccinated mice.

## Results

### The rBCG-pMyong2-p24 Strain Elicited Enhanced HIV-1 p24 Gag Expression in Bacteria and Infected Cells

**Figure 1:**
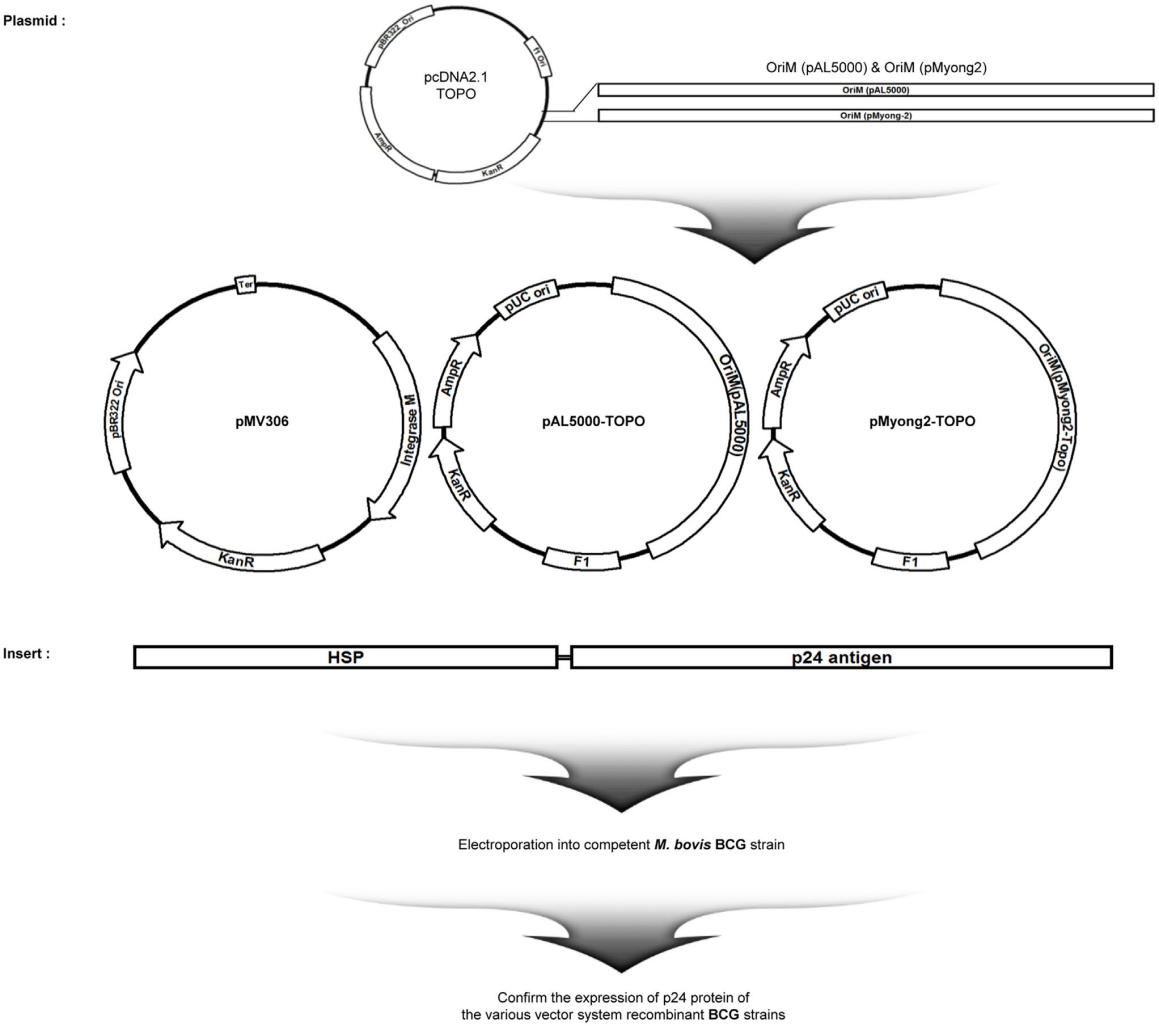
Maps of p24 expression vectors of the study. Maps of the constructed p24 expression *Mycobacterium–Escherichia coli* shuttle vectors. pMV306-p24, pALp24, and pMyong2-p24 vectors expressed p24 under control of the *hsp65* promoter *from M. bovis* BCG.

**Figure 2:**
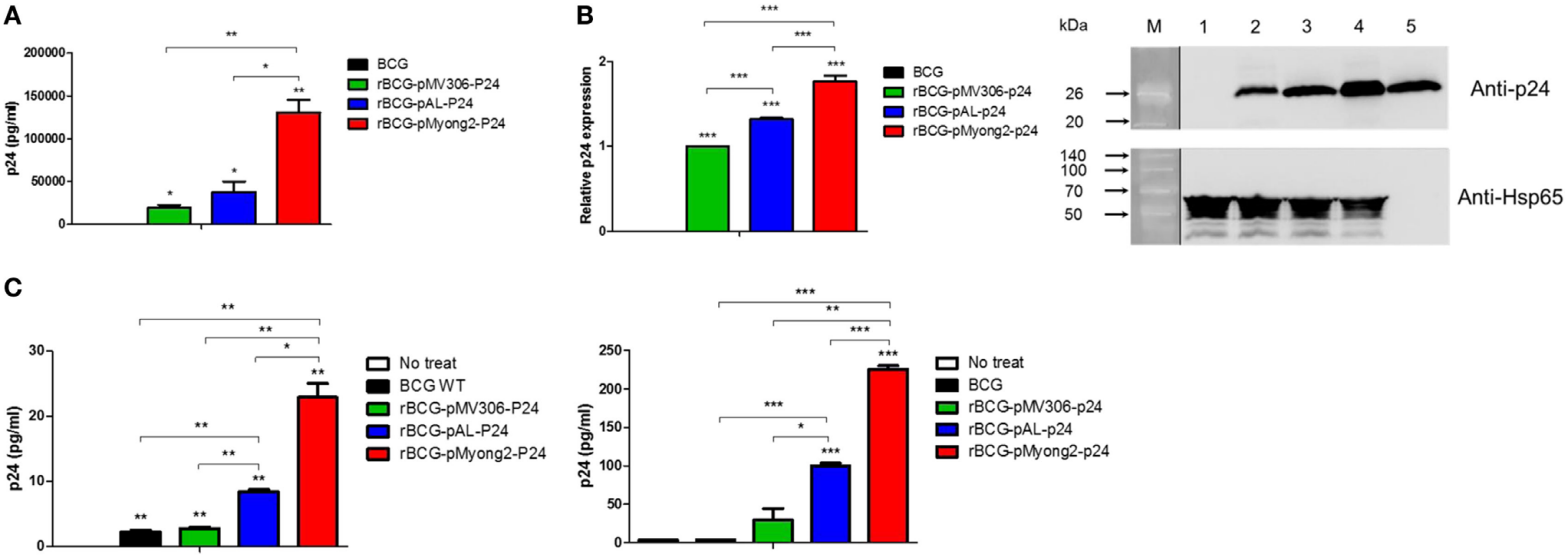
Expression levels of p24 in recombinant *Mycobacterium bovis* BCG (rBCG) strains and cell lines infected with rBCG strains. **(A)** Confirmation of p24 expression in rBCG strains using enzyme-linked immunosorbent assay. **(B)** Confirmation of p24 expression in rBCG strains using a Western blot analysis. Proteins were extracted from wild-type BCG (lane 1) and rBCG strains (lane 2, rBCG-pMV306-p24; lane 3, rBCG-pAL-p24; and lane 4, rBCG-pMyong2-p24). Purified p24 protein was used as a positive control (lane 5). M, molecular weight standard (Elpis Bio, Taejeon, South Korea; DokDo-MARK™). Distinct membranes are separated by white space. In addition, the marker lane is separated by a vertical black line. The expression levels of p24 are plotted. The Western blot image was cropped from a full-length blot to improve clarity. The full-length blot image is presented in the Figure S5 in Supplementary Material. **(C)** The expression levels of p24 after the infection of the murine macrophage cell line J774A.1 (left panel) and bone-marrow derived dendritic cells (right panel) with wild-type BCG and rBCG strains (rBCG-pMV306-p24, -pAL-p24, and -pMyong2-p24). Data are representative of two independent experiments. Mean ± SD are shown. *P < 0.05; **P < 0.01; ***P < 0.001 (Student’s *t*-test).

To examine the usefulness of the pMyong2 vector system in the generation of rBCG for HIV-1 p24 Gag vaccination, we generated three types of rBCG strains expressing p24, i.e., rBCG-pMyong2-p24, rBCG-pAL-p24, and rBCG-pMV306-p24, using different *Mycobacterium–E. coli* shuttle vectors, i.e., pMyong2-TOPO, pAL-TOPO (39), and pMV306 (46), respectively (Figure 1). The growth rates of the three rBCG strains in the 7H9 broth (with 100 µg/ml of kanamycin) for 30 days were compared, and the rBCG and wild-type BCG strains showed a nearly identical growth rate (Figure S1 in Supplementary Material). Additionally, to examine the survival of these rBCG strains in the macrophages and DCs, the infected cells were lysed with 0.05% Triton X-100 (in PBS) and plated onto 7H10 agar plates. In both cells, the rBCG-pMyong2-p24 strain had fewer colony forming units (CFUs) than the other strains (i.e., rBCG-pAL-p24, -pMyong2-p24, and wild-type BCG strains) (Figure S2 in Supplementary Material) likely due to the bacterial burden by the enhanced p24 expression (40). To compare the expression levels of p24 in the bacteria of the three rBCG strains, we conducted enzyme-linked immunosorbent assay (ELISA) (Figure 2A) and Western blot (Figure 2B) analyses against p24 after lysis of the cultured bacteria. All rBCG strains could express the p24 protein. Similar to the rSmeg-pMyong2-p24 strain (40), the rBCG-pMyong2-p24 strain expressed approximately two or three times higher levels of p24 than the strains in the other vector systems. rBCG-pAL-p24 produced a slightly higher level of p24 than rBCG-pMV306-p24 (Figures 2A,B). To assess the stable expression of p24, the expression levels of p24 in rBCG-pMyong2-p24 in various passage points on 7H10 agar plates with or without kanamycin were also determined by Western blot analyses. The rBCG-pMyong2-p24 strain showed stable p24 expression even after 12 passages on the 7H10 agar plates with or without kanamycin (Figures S3 and S4 in Supplementary Material). Additionally, we examined the expression levels of p24 by ELISA in infected murine macrophages (J774A.1) and BMDCs infected with three rBCG strains. The trends observed were similar to those observed with the lysed rBCG strains (Figure 2C). Also, to compare the p24 expression levels according to the different M.O.I., BMDCs were infected with different M.O.I. (1 and 10 M.O.I.) of rBCG-pAL-p24 and rBCG-pMyong2-p24 for 1 and 3 days. The results showed that the increased M.O.I. of both strains affected the increased p24 expression. However, as presented above, rBCG-pMyong2-p24 induced more p24 expression than rBCG-pAL-p24 strain (Figure S8 in Supplementary Material). Altogether, compared to the other two rBCG strains, i.e., rBCG-pAL-p24 and rBCG-pMV306-p24, rBCG-pMyong2-p24 increased the production of p24 in infected antigen-presenting cells and bacteria.

### BMDCs Infected With the rBCG-pMyong2-p24 Strain Elicited Enhanced T Cell Proliferation in Mice Immunized With HIV-1 p24 Gag

**Figure 3:**
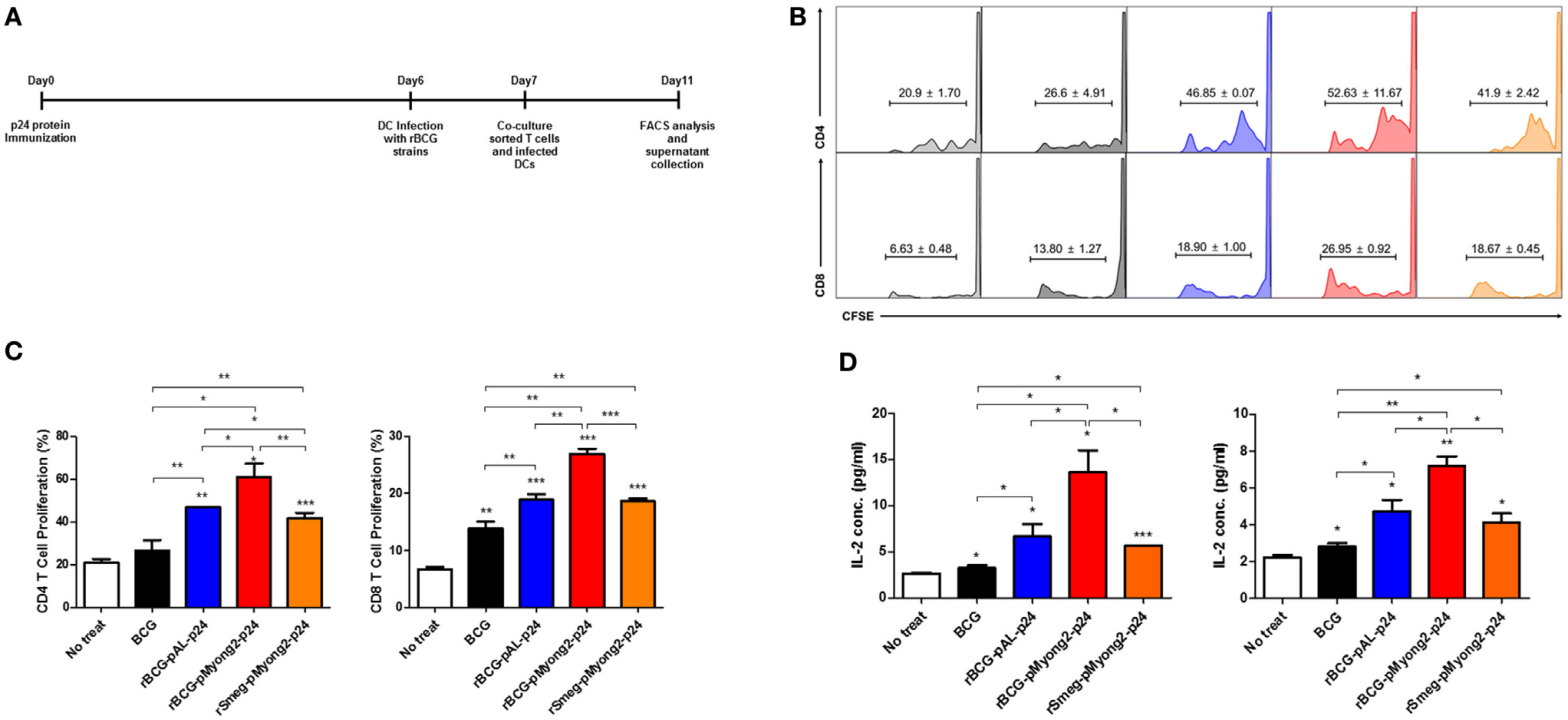
T cell proliferation levels induced by bone-marrow derived dendritic cells (BMDCs) infected with the p24 recombinant *Mycobacterium bovis* BCG (rBCG) strains. **(A)** Schematic schedule of the T cell proliferation assay. Two mice were injected with p24 protein (30 μg/mouse) and after 7 days, their splenocytes were sorted into CD4 and CD8 T cells and labeled with CFSE. One day before co-cultivation, DCs were infected with each strain (10 M.O.I.). Four days after co-cultivation of CFSE-labeled CD4/CD8 T cells and infected DCs, cells were analyzed for T cell proliferation. **(B,C)** Flow cytometric analysis of the proliferation of CFSE-labeled CD4 and CD8 T cells following the infection of BMDCs with the p24 rBCG strains. **(D)** Enzyme-linked immunosorbent assay determination of IL-2 released in the supernatants of CD4 (left panel) and CD8 (right panel) cells using a mixed lymphocyte response assay. Data are representative of three independent experiments. Mean ± SD are shown. *P < 0.05; **P < 0.01; ***P < 0.001 (Student’s *t*-test).

To determine whether the rBCG-pMyong2-p24 showing enhanced p24 protein production improved the T cell proliferation capacity, we conducted a T cell proliferation assay in BMDCs infected with four different strains, i.e., a wild-type BCG (as a control), two types of rBCG (rBCG-pMyong2-p24 and rBCG-pAL-p24), and rSmeg-pMyong2-p24 (40), *via* mixed lymphocyte response using CFSE dilution methods (47). The rSmeg-pMyong2-p24 strain was also included to compare the capacity of inducing HIV-1 p24 Gag-specific immune responses between two different species using the same pMyong2 vector system, i.e., rBCG-pMyong2-p24 and rSmeg-pMyong2-p24. A schematic of the T cell proliferation assay is shown in (Figure 3A). All BMDCs infected with the two rBCG and one rSmeg strains induced significantly higher levels of CD4 and CD8 T cell proliferation than the BMDCs that were not infected. Notably, the BMDCs infected with rBCG-pMyong2-p24 induced significantly higher levels of CD4 and CD8 T cell proliferation than those infected with the other two recombinant strains, i.e., rBCG-pAL-p24 and rSmeg-pMyong2-p24 strains, and the wild-type BCG strain. However, no significant difference was observed between the BMDCs infected with rBCG-pAL-p24 and those infected with rSmeg-pMyong2-p24 in the proliferation of both CD4 and CD8 T cells (Figures 3B,C). The comparison of the IL-2 levels in stimulated CD4 and CD8 T cells also showed trends that were similar to those observed in T cell proliferation assays (Figure 3D).

### The rBCG-pMyong2-p24 Strain Elicited Enhanced HIV-1 p24 Gag-Specific IFN-γ Spot Forming Cells in Mice Spleens Generated by Subcutaneous Immunization

**Figure 4:**
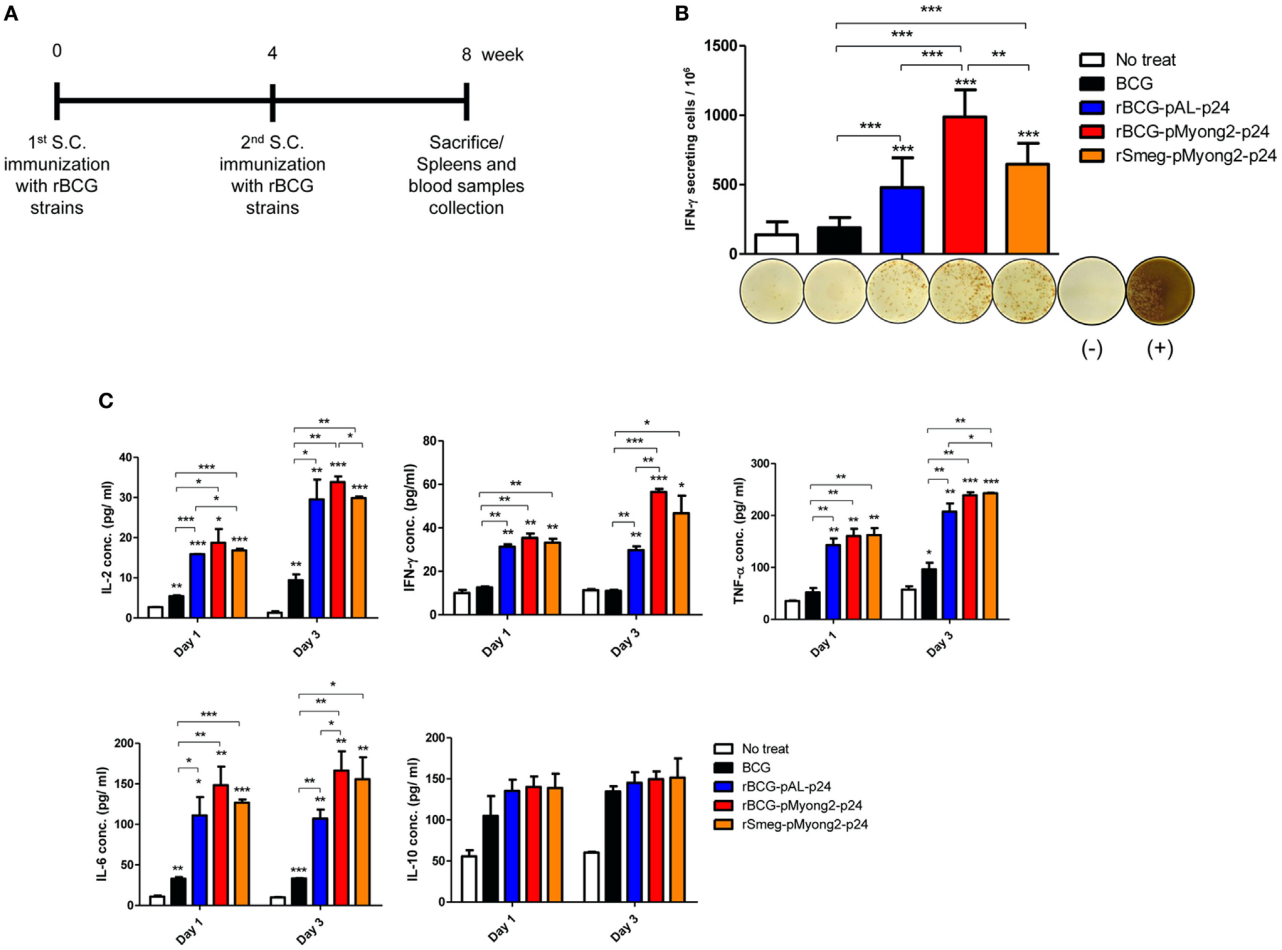
*In vivo* immune responses induced by p24 recombinant *Mycobacterium bovis* BCG (rBCG) strains. **(A)** Schematic of the immunization performed for the *in vivo* immunological assays. With 4 weeks interval, each group (five mice/group) was immunized twice with wild-type BCG, two rBCG strains, and rSmeg strain. Four weeks after final immunization, mice were sacrificed and their spleens and blood samples were collected for immunological analyses. **(B)** IFN-γ secretion levels following *in vitro* stimulation of splenocytes from mice vaccinated with the p24 rBCG strains were detected using an ELISPOT analysis. Representative images of ELISPOT membrane in each group are shown below the graph. (−), negative control; (+), positive control. **(C)** Levels of the IL-2, IFN-γ, TNF-α, IL-6, and IL-10 cytokines following *in vitro* stimulation with p24 of splenocytes from mice vaccinated with the p24 rBCG strains were detected using enzyme-linked immunosorbent assay analyses. A total of five mice per group was analyzed. Data are representative of two independent experiments. Mean ± SD are shown. *P < 0.05; **P < 0.01; ***P < 0.001 (Student’s *t*-test).

To determine whether rBCG-pMyong2-p24 improved the T cell response after vaccination, splenocytes were isolated from the spleens of BALB/c mice (five mice/group) that were subcutaneously (s.c.) immunized with three different strains, i.e., two types of rBCG strains (rBCG-pMyong2-p24 and -pAL-p24), rSmeg-pMyong2-p24 (Figure 4A), and a wild type BCG strain as a control (~10^6^ CFU) and assayed for HIV-1 p24 Gag-specific T cell responses using IFN-γ ELISPOT assays. The splenocytes from the mice that were s.c. immunized with three recombinant strains showed significantly higher spot forming units (SFUs) than those from the mice that were immunized with the wild-type BCG strain. Notably, the splenocytes from mice immunized with rBCG-pMyong2-p24 (987.78 ± 195.11 SFUs/10^6^ splenocytes) yielded significantly higher SFUs than those from mice immunized with the other two strains, i.e., rBCG-pAL-p24 (479.56 ± 213.90 SFUs/10^6^ splenocytes) and rSmeg-pMyong2-p24 (647.00 ± 151.01 SFUs/10^6^ splenocytes) (Figure 4B). However, no significant difference was observed between the rBCG-pAL-24 and rSmeg-pMyong2-p24 strains in p24-specific IFN-γ SFUs from vaccinated mice (Figure 4B). Altogether, our data indicated that rBCG-pMyong2-p24 elicited an enhanced HIV-1 p24 Gag-specific production of IFN-γ, which is a Th-1 signature cytokine, suggesting its feasibility for enhancing vaccine efficacy by skewing the Th-1 type immune responses.

### The rBCG-pMyong2-p24 Strain Elicits an Enhanced Production of Th1 or Pro-Inflammatory Cytokines in Splenocytes From Vaccinated Mice

The splenocytes (five mice/group) obtained 4 weeks after the second immunization with the rBCG strains and rSmeg-pMyong2-p24 (Figure 4A) were stimulated *in vitro* in triplicate with purified p24 protein (5 µg/ml), and the induced cytokine productions of IL-2, IFN-γ, TNF-α, IL-6, and IL-10 in the cell culture supernatants were detected. In two Th1 type cytokines, i.e., IL-2 and IFN-γ, and one pro-inflammatory cytokine, i.e., IL-6, the rBCG-pMyong2-p24 strain always produced higher level of cytokines in splenocytes from vaccinated mice at all-time points (day 1 and 3) than the wild type or other two recombinant strains. (Figure 4C; Table S1 in Supplementary Material). Regarding the other pro-inflammatory cytokine, i.e., TNF-α, the rBCG-pMyong2-p24 strain produced higher level of cytokines than rBCG-pAL-p24, but an almost identical level of TNF-α was observed in both the rBCG-pMyong2-p24 and rSmeg-pMyong2-p24 strains. In the case of IL-10, which is a Th-2 cytokine, all three recombinant strains showed similar secretion levels (Figure 4C; Table S1 in Supplementary Material).

### The rBCG-pMyong2-p24 Strain Elicits an HIV-1 p24 Gag-Specific Th1-Biased Humoral Response in Immunized Mice

**Figure 5:**
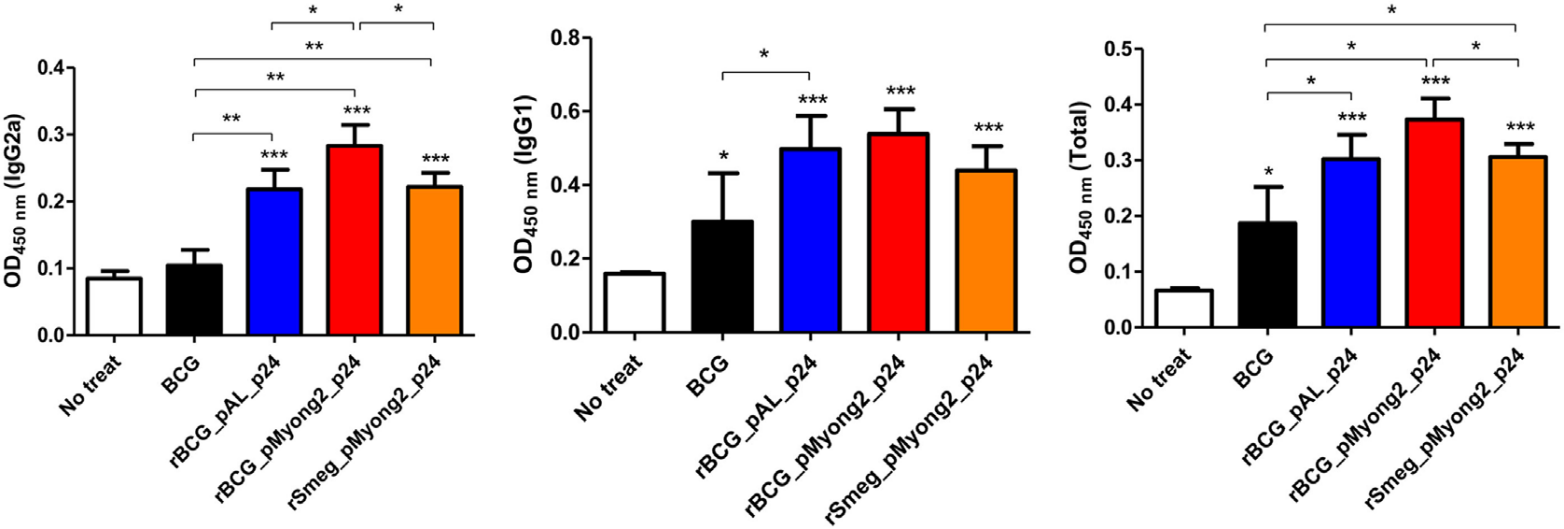
Humoral immune responses induced by p24 recombinant *Mycobacterium bovis* BCG (rBCG) strains. p24-specific immunoglobulin subtypes (IgG2a, IgG1, and total IgG) were detected by enzyme-linked immunosorbent assay at 450 nm. Optical density (OD) values for the IgG2a and IgG1 subtypes and the ratio of IgG2a/IgG1 were compared. Serum samples from five mice per group were analyzed. Data are representative of two independent experiments. Mean ± SD are shown. *P < 0.05; **P < 0.01; ***P < 0.001 (Student’s *t*-test).

To determine whether rBCG-pMyong2-p24 elicits a Th1-biased humoral response in immunized mice, we analyzed the levels of HIV-1 p24 Gag-specific IgG2a and IgG1, which are known markers of Th1 and Th2 responses, respectively (48–50). As shown in Figure 5, the two rBCG and rSmeg-pMyong2-p24 strains elicited significantly higher levels of IgG2a isotype than the wild type. Regarding the IgG1 isotype, the three recombinant strains elicited similar level of IgG1; however, the result did not reach statistical significance. In the case of total IgG, rBCG-pMyong2-p24 showed significantly higher levers of total IgG than the other two recombinant strains (i.e., rBCG-pAL-p24 and rSmeg-pMyong2-p24) (Figure 5). Collectively, the IgG2a/IgG1 ratio, in which a higher ratio indicates a more Th1-biased humoral immune response (49), was higher in the sera from the mice immunized with rBCG-pMyong2-p24 (1.03 ± 0.02) than that in the sera from the mice immunized with the other strains (wild-type BCG = 0.91 ± 0.71; rBCG-pAL-p24 = 0.88 ± 0.21; rSmeg-pMyong2-p24 = 1.01 ± 0.17), suggesting that the rBCG-pMyong2-p24 strain can elicit an enhanced HIV-1 p24 Gag-specific Th1-biased humoral response in immunized mice.

### The rBCG-pMyong2-p24 Strain Elicits an Enhanced HIV-1 p24 Gag-Specific Cytotoxic T Lymphocyte Response in Immunized Mice

**Figure 6:**
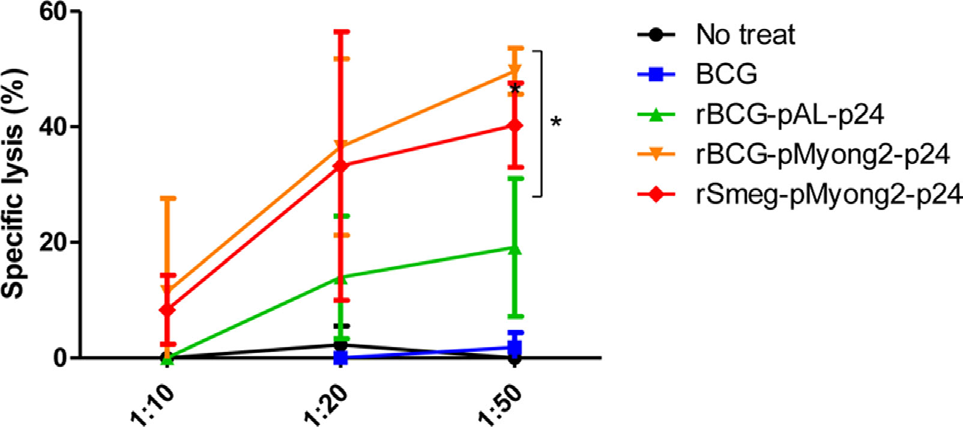
Cytotoxic T lymphocyte responses in mice immunized with the recombinant *Mycobacterium bovis* BCG (rBCG) strains. CTL responses due to the reaction of *in vitro* stimulated splenocytes with p24 (effector cells) and p24 epitope peptide (A9I) pulsed P815 cells (target cells). Three mice per group were analyzed. Data are representative of two independent experiments. Mean ± SD are shown. *P < 0.05 (Student’s *t*-test).

To determine whether rBCG-pMyong2-p24 elicits an enhanced HIV-1 p24 Gag-specific cytotoxic T lymphocyte (CTL) response in immunized mice, we analyzed the CTL activity in splenocytes from mice immunized with two rBCG (i.e., rBCG-pMyong2-p24 and -pAL-p24), rSmeg-pMyong2-p24, or wild-type BCG strains *via* a lactate dehydrogenase (LDH) cytotoxicity assay. The immunization procedure is described in Figure 4A. The P815 cells (H-2^d^) that were pulsed with the A9I peptide for 2 h served as the target cells, and the effector/target ratios were 10:1, 20:1, and 50:1 as previously described (40). As shown in Figure 6, at an E:T ratio of 50:1, the CTLs in the mice immunized with rBCG-pMyong2-p24 could elicit a significant higher level of HIV-1 p24 Gag-specific target cell lysis than those immunized with other strains (Figure 6). However, no significant difference was observed between the rBCG-pMyong2-p24 and rSmeg-pMyong2-p24 strains (Figure 6).

### The rBCG-pMyong2-p24 Strain Elicits Enhanced HIV-1 p24 Gag-Specific Humoral and Cell-Mediated Immune Responses in Immunized Mice, Compared to p24 Protein Vaccination

**Figure 7:**
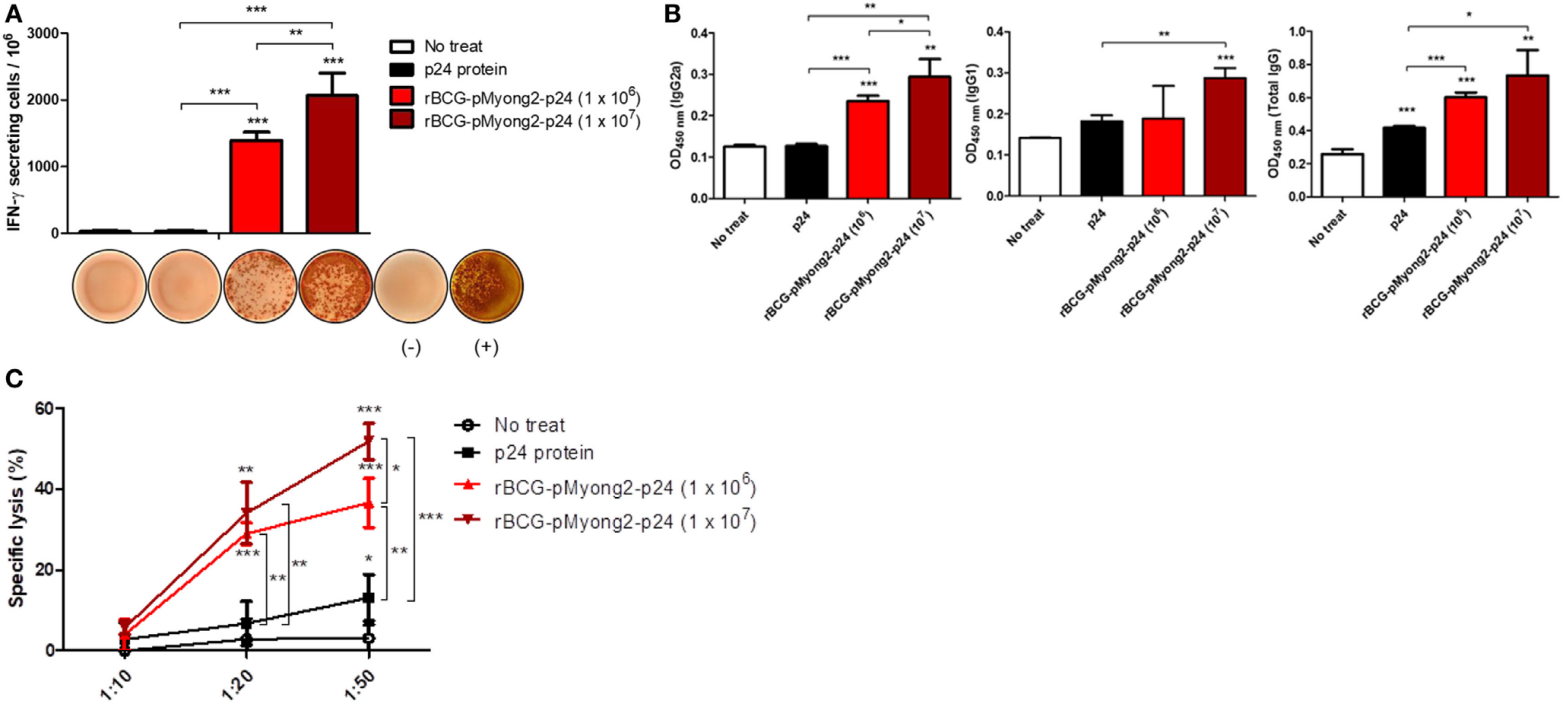
Comparison of the p24-specific immune responses by injections of p24 protein and different M.O.I. of recombinant *Mycobacterium bovis* BCG (rBCG)-pMyong2-p24 strains. **(A)** Comparison the IFN-γ secretion levels following *in vitro* stimulation of splenocytes from mice (three mice/group) injected subcutaneously with the p24 protein (30 μg/mouse) and different colony forming units (CFUs) (1 × 10^6^ and 1 × 10^7^ CFU) of rBCG-pMyong2-p24 strain (1 week interval, twice injection) were detected using an ELISPOT analysis. Representative images of ELISPOT membrane in each group are shown below the graph. (−), negative control; (+), positive control. Data are shown with Mean ± SD in triplicate. **P < 0.01; ***P < 0.001 (Student’s *t*-test). **(B)** p24-specific immunoglobulin subtypes (IgG2a, IgG1, and total IgG) were detected by enzyme-linked immunosorbent assay. Serum samples from three mice per group were analyzed. Data are shown with Mean ± SD in triplicate. *P < 0.05; **P < 0.01; ***P < 0.001 (Student’s *t*-test). **(C)** Cytotoxic T lymphocyte responses due to the reaction of splenocytes (stimulated with A9I, p24 epitope peptide; effector cells) from p24 protein and rBCG-pMyong2-p24 injected mice and A9I peptide pulsed P815 cells (target cells). Three mice per group were analyzed. Data are shown with Mean ± SD in triplicate. *P < 0.05; **P < 0.01; ***P < 0.001 (Student’s *t*-test).

To compare the p24-specific immune responses between p24 protein and different CFUs of rBCG-pMyong2-p24 strains, we conducted an independent *in vivo* experiment with immunization groups of (i) PBS control, (ii) p24 protein (30 μg/mice) injection, (iii) rBCG-pMyong2-p24 (1 × 10^6^ CFU) injection, and (iv) rBCG-pMyong2-p24 (1 × 10^7^ CFU) injection (1 week interval, twice S.C. injection). The immunization procedure is described in Section “Materials and Methods.” After final immunization, we conducted p24-specific IFN-γ ELISPOT, IgG subtype analyses, and CTL analyses. In the case of IFN-γ ELISPOT analysis, the p24-specific IFN-γ SFUs were increased in a CFU-dependent manner. However, splenocytes from p24 protein injected mice could not induce the p24-specific IFN-γ SFUs (Figure 7A). Similarly, the p24-specific IgG2a antibodies in serum samples from each immunized mouse were also increased in a CFU-dependent manner. However, the p24-specific IgG2a antibody from serum of p24 protein injected mice showed lower levels, compared to those of rBCG-pMyong2-p24 injection groups (Figure 7B). Also, we compared the p24-specific CTL responses between p24 protein and different CFUs of rBCG-pMyong2-p24 strains. Our data showed that p24-specific CTL responses of rBCG-pMyong2-p24 were increased in a CFU-dependent manner and were always significantly higher than those of p24 protein (Figure 7C). Collectively, our data suggest that rBCG-pMyong2-p24 could elicit p24-specific Th1-biased cellular and humoral immune responses in a CFU-dependent manner and may have a merit as an HIV-1 vaccine regimen, compared to p24 protein vaccination module.

## Discussion

The best strategy reported to improve the potential of rBCG as an HIV vaccine is its use as a prime vaccine in a prime-booster vaccine protocol using a safe recombinant viral vector for a booster vaccine, which has been shown to induce a long-lasting effective virus-specific cellular immunity after vaccination in animal models (28). In this context, the Th1 response induced by the rBCG vector may contribute to eliciting the Gag-specific CTL response (51). However, the major barrier to the practical use of rBCG as an HIV-1 vaccine is the failure to elicit sufficient virus-specific CTL responses to protect against virus infection due to the low expression of the foreign HIV-1 antigens in rBCG (28, 29). To overcome this limitation, several strategies, including the use of a hemolysin-expressing BCG strain capable of eliciting a greater CTL response *via* the preferential cytosol location of rBCG (51) and a codon optimization for the HIV-1 gag p24 gene in the rBCG system (52) have been introduced. In this study, we applied the pMyong2 shuttle vector system to enhance the expression of the HIV-1 gag p24 gene in the rBCG, which has already been shown to be useful in rSmeg in our previous study (40). We found that rBCG-pMyong2-p24 produced more P24 proteins in the infected macrophages and BMDCs than the conventional episomal pAL5000 vector (rBCG-pAL-p24) and integrating pMV306 vector (rBCG-pMV306-p24) (Figure 2), providing a mechanistic basis of the enhanced virus-specific vaccine efficacy of rBCG-pMyong2-p24, including enhanced p24-specific T cell proliferation of BMDCs (Figures 3B,C), T cell effector function (Figures 4B,C), particularly in CTLs (Figure 6), and Th1-biased humoral immune response (Figure 5). Previously, we showed that rSmeg-pMyong2-p24 was more attenuated in macrophage infection than rSmeg strains using other vector systems. Indeed, a similar trend was also observed in rBCG-pMyong2-p24, which produced a lower level of CFUs than rBCG-pAL-p24 (Figure S2 in Supplementary Material) likely due to higher copy numbers of pMyong2 in rBCG than those in the other vector systems. Given the previous findings in which immunization with a lower dose or more attenuated rBCG could reduce the risks associated with high-dosage cutaneous administration, including adverse local skin reactions, possible association with Th2-type immune responses, or exacerbation of retroviral infections (52–54). rBCG-pMyong2-p24 could have an additive advantage in HIV vaccine protocols using rBCG.

Compared with integrative plasmid systems, multi-copy episomal vector-based *Mycobacterium*–*E. coli* shuttle vector systems have been reported to have a drawback regarding the lack of stability of the recombinant *Mycobacterium* (4, 29, 55). Indeed, our previous study showed that the pMyong2-p24 plasmid gradually lost its stability in rSmeg (rSmeg-pMyong2-p24) after 5 passages in media without antibiotics (40). However, surprisingly, despite using the same pMyong2 vector system, we found that the pMyong2-p24 plasmid could maintain its stability in rBCG (rBCG-pMyong2-p24), even after 12 passages regardless of whether antibiotics were added (Figure S4 in Supplementary Material), suggesting that the pMyong2 plasmid from the slow growing *M. yongonense* may be more stable in slow growing mycobacteria, such as BCG, than in rapid growing mycobacteria, such as Smeg. Given that the stability of incorporating a plasmid in antibiotic free media is pivotal to recombinant live vaccine production for practical use, rBCG-pMyong2-p24 likely has an advantage over rSmeg-pMyong2-p24 in application as an HIV-1 vaccine.

In this study, in addition to using rBCG strains in different episomal vector systems, i.e., rBCG-pMyong2-p24 and rBCG-pAL-p24, we also compared the vaccine efficacy against HIV-1 in two different mycobacteria, i.e., BCG (rBCG-pMyong2-p24) and Smeg (rSmeg-pMyong2-p24), using the same pMyong2 system. To the best of our knowledge, this is the first comparison of rBCG and rSmeg for vaccine efficacy. In immune responses against HIV-1 p24 antigens, although the CTL response, T cell proliferation capacity of infected BMDC and most IFN-γ ELISPOT level from immunized splenocytes were almost identical in rBCG-pMyong2-p24 and rSmeg-pMyong2-p24, the former showed a significantly enhanced IL-2 production in splenocytes and Th1-biased humoral immune responses compared to the latter, suggesting that the former may be superior to the latter in HIV-1 vaccine regimens.

In addition, we also compared the vaccine efficacy against HIV-1 between two different vaccine modules, using rBCG-pMyong2-p24 and p24 proteins. Our data indicated that rBCG-pMyong2-p24 had the enhanced p24-specific IFN-γ ELISPOT level, CTL response, and Th1-biased humoral immune response, compared to p24 proteins (Figure 7), also suggesting that the former may be superior to the latter in HIV-1 vaccine regimens.

It has been reported that there is a gender disparity in response to diverse vaccines, including BCG, the measles, mumps and rubella vaccine, and influenza vaccines (56). Generally, in adaptive immune responses, females exhibit enhanced humoral and cell-mediated immune responses, compared to males (57). This is the reason why we selected only female mice for the current vaccine study. Further evaluation of rBCG-pMyong2-p24 in HIV-1 vaccine efficacy using male mice should be needed for the future.

In the current study, we demonstrated that rBCG-pMyong2-p24 in the pMyong2 vector system elicited higher levels of HIV-1 p24 Gag protein expression in rBCG and delivered more p24 antigens into phagocytes than the other BCG strains using the pAL5000- (rBCG-pAL-p24) or pMV306-derived system (rBCG-pMV306-p24). We also showed that this strain could enhance the T cell proliferation capacity of infected BMDCs and elicit improved CTL responses and Th1-biased humoral immune responses in vaccinated mice, compared to rBCG-pAL-p24 or rSmeg-pMyong2-p24. These findings suggest that rBCG-pMyong2-p24 may be an effective candidate as a prime vaccine in a heterologous prime-boost vaccine strategy for HIV-1 infection.

## Materials and Methods

### Mice and Immunization Procedures

Female BALB/c mice (~25 g, 7 weeks old) were purchased from Orient-Bio (Seoul, South Korea) and used in the experiments at the age of 8 weeks. The mice were randomly divided into four groups of five mice per group.

For T cell proliferation assay, p24 protein was injected into two mice (BALB/c) through the tail vein (30 μg/mouse) and five mice (BALB/c) was used for preparation of BMDCs in each test.

For vaccination test, the BALB/c mice were subcutaneously immunized with wild type, two rBCG strains (i.e., rBCG-pAL-p24 and rBCG-pMyong2-p24), or the rSmeg-pMyong2-p24 strain twice (1 × 10^6^ CFU in the 100 µl PBS; at a 4-week interval) at the bottom of the tail. For negative control group, PBS was injected subcutaneously. After 4 weeks after the final immunization, the mice were euthanized at each time point by CO_2_ inhalation, and their bloods and spleens were removed and used in the immunological assays, such as IFN-γ ELISPOT, cytokine determination, serum antibody detection (five mice/group), and CTL analysis (three mice/group).

Also, an independent *in vivo* test was conducted to compare the difference in immune responses induced by p24 protein treat and different bacterial number. In this case, with 1 week interval, the BALB/c mice (three mice/group) were subcutaneously injected p24 protein (30 μg/mice) and different number of rBCG-pMyong2-p24 strain (1 × 10^6^ and 1 × 10^7^ CFU) two times. For negative control group PBS was injected subcutaneously. One week after the final immunization, the mice were euthanized at each time point by CO_2_ inhalation, and their bloods and spleens were removed and used in the immunological assays, such as IFN-γ ELISPOT, serum antibody detection, and CTL analysis.

### Ethics Statement

All animal experiments were carried out in accordance with the recommendations of institutional guidelines and the protocol approved by the Institutional Animal Care and Use Committee (approval No. of SNU-160307-1-2) of the Institute of Laboratory Animal Resources at Seoul National University.

### Generation of rBCG Strains Expressing HIV-1 p24 Gag

To generate three different types of rBCG strains expressing HIV-1 p24 Gag, BCG with the pMyong2-p24 plasmid (designated rBCG-pMyong2-p24), BCG with the pAL-p24 plasmid (designated rBCG-pAL-p24), and BCG with the pMV306-p24 plasmid (designated rBCG-pMV306-p24), the three constructed plasmids, i.e., pMV306-p24, pAL-p24, and pMyong2-p24 (40), were electroporated into the competent BCG strain (Tokyo 172) using the Gene Pulser II electroporation apparatus (Bio-Rad, Hercules, CA, USA) (58). Transformants were selected on Middlebrook 7H10 medium (Difco Laboratories, Detroit, MI, USA) supplemented with OADC containing 100 µg/ml of kanamycin. Typically, colonies of transformants were selected from the plates, transferred into 7H9 broth medium (Difco Laboratories, Detroit, MI, USA) supplemented with 0.5% glycerol, 0.05% Tween-80, 10% ADC and kanamycin and cultured for 3~4 weeks. The growth rate of the rBCG strains was determined by optical density (OD) at 600 nm.

### Production of p24 Protein From *E. coli*

Recombinant p24 proteins were purified from *E. coli* as previously described (59) with minor modification. For the expression and purification of the fusion protein, *E. coli* BL21 strains (RBC Bioscience, Taipei City, Taiwan) were transformed with pET23a-p24. Protein expression was induced by adding 0.4 mM isopropyl β-d-thiogalactoside (Duchefa Biochemie, Haarlem, Netherlands). Bacterial cells were harvested and disrupted by sonication on ice for 10 min. Sonicated lysates were centrifuged at 1,600 × *g* for 20 min at 4°C, and the pellets containing p24 protein were resuspended in binding buffer containing 4 M urea (Sigma Aldrich, St. Louis, MO, USA). The proteins were purified using Ni-NTA His binding resin (Merck, Darmstadt, Germany) and eluted with elution buffer (300 mM NaCl, 50 mM sodium phosphate buffer, 250 mM imidazole) containing 4 M urea. Purified proteins were dialyzed serially against the elution buffer to remove imidazole, urea, and residual salts. Purity of p24 protein was estimated by sodium dodecyl sulfate-polyacrylamide gel electrophoresis (SDS-PAGE; 12% gel). The gel was visualized using Coomassie brilliant blue staining methods (60) (Figure S9 in Supplementary Material).

### Generation of BMDCs From Mice

Bone marrow-derived dendritic cells were generated from the bone marrow (BM) of 8- to 12-week-old BALB/c mice as previously described (40). Briefly, the BM cells were flushed out of the femurs and tibias with serum-free Iscove’s modified Eagle medium (IMDM; Gibco Invitrogen, UK). The single cell suspension was seeded at a concentration of 1 × 10^6^ cells per well in a 24-well plate in final volume of 2 ml of complete IMDM supplemented with 10% fetal bovine serum (FBS) (Gibco Invitrogen), recombinant mouse GM-CSF (1.5 ng/ml; PeproTech, Rocky Hill, NJ, USA) and mouse IL-4 (1.5 ng/ml; PeproTech, USA), penicillin (100 units/ml; Gibco Invitrogen), streptomycin (100 µg/ml; Gibco Invitrogen), gentamicin (50 µg/ml; Gibco Invitrogen), l-glutamine (2 mM; Gibco Invitrogen), and β-mercaptoethanol (50 nM; Gibco Invitrogen). Half of the medium was replaced every other day with an equal volume of complete IMDM for 6 days. Five mice were used to prepare each experiment using BMDCs and five 24-well plates were used for differentiating the BMDCs.

### CFU Assay in Infected J774A.1 and BMDCs With rBCG Strains

The murine macrophage cell line J774.1 (American Type Culture Collection, TIB-67) was maintained at 37°C and 5% CO_2_ in Dulbecco’s modified Eagle’s medium (Thermo Scientific, Rockford, IL, USA) supplemented with 10% (v/v) FBS, 2 mM glutamine, and essential amino acids. BMDCs were generated from mouse BM as previously described (40, 61) and maintained at 37°C and 5% CO_2_ in Iscove’s modified Eagle medium (IMDM; Gibco Invitrogen, UK) supplemented with 10% FBS (Gibco Invitrogen), recombinant mouse GM-CSF (1.5 ng/ml; PeproTech, Rocky Hill, NJ, USA), mouse IL-4 (1.5 ng/ml; PeproTech, USA), penicillin (100 units/ml; Gibco Invitrogen), streptomycin (100 µg/ml; Gibco Invitrogen), gentamicin (50 µg/ml; Gibco Invitrogen), l-glutamine (2 mM; Gibco Invitrogen), and β-mercaptoethanol (50 nM; Gibco Invitrogen). The J774A.1 cells and BMDCs were infected with the rBCG strains, rBCG-pMyong2-p24, -pAL-p24, and -pMV306-p24 and wild type BCG strains (10 M.O.I.) (in triplicate) for 4 h, followed by three washes with PBS and culturing for 24 h with fresh media. After 24 h, the infected cells were lysed with 0.5% Triton X-100. The cell lysates were diluted with PBS and plated onto Middlebrook 7H10 agar plates supplemented with OADC for enumeration of the CFUs. All the infection groups were analyzed in triplicates in each experiment, and total two independent experiments were conducted.

### Determination of the p24 Gag Expression Levels in the rBCG Strains

To determine the p24 Gag expression levels in the rBCG strains, we conducted Western blot and ELISA analyses as previously described (40). Briefly, pellets of cultured rBCG strains were suspended in B-PER buffer (Thermo scientific, Rockford, IL, USA) supplemented with lysozyme (100 µg/ml), DNase (5 U/ml), and a proteinase inhibitor. Then, the suspensions were sonicated for 5 min (pulse: 0.3 s, stop: 0.7 s) on ice and centrifuged at 13,000 rpm at 4°C for 15 min. The same amount of proteins in the aqueous phase was used for the Western blot analysis. The expression levels of p24 in each rBCG strain were determined using a mouse anti-p24 monoclonal antibody (Abcam, Cambridge, MA, USA; 1:1,000 dilution). Mycobacterial Hsp65 (Abcam, 1:1,000 dilution) was used as an internal control to confirm that the protein concentrations were equal in all samples. To assess the stable expression of p24, the p24 expression level in the rBCG-pMyong2-p24 strain of various passage points (after 1, 4, 6, 8, 10, and 12 passages) was also determined. The passage process was conducted from plate to plate (7H10 agar plate with or without kanamycin), and the colonies from each passage were cultured in 7H9 broth medium for 3 weeks prior to performing each of the experiments. Additionally, the same amount of proteins was used for the detection of the p24 levels using the p24 ELISA kit (in triplicate well) (ABL, Rockville, MD, USA) as suggested by the manufacturer (62). All the groups were analyzed in two independent experiments.

### Determination of the p24 Gag Expression Levels in BMDCs and J774.1 Cells Infected With the rBCG Strains

For the rBCG infection, the J774.1 cells and BMDCs were seeded 5~10 × 10^5^ cells per well (24-well plate, in triplicate) and cultured for 18 h. The three different rBCG strains were infected into the cells at a multiplicity of infection (M.O.I) of 10. Also, different M.O.I (1 and 10 M.O.I) of rBCG-pMyong2-p24 strain was infected into BMDCs to compare the difference in p24 expression by different M.O.I. The J774.1 cells and BMDCs were incubated for 4 h to allow phagocytosis of the bacteria, and the extracellular bacteria were removed by washing with PBS three times. The infected J774.1 cells and BMDCs were incubated for 24 h and/or 72 h. For analysis the p24 expression in the cells, the total proteins in the cell pellets were prepared by suspension in RIPA lysis buffer and used for the determination of the p24 levels using the p24 ELISA kit (ABL) (in triplicate well) according to the manufacturer’s instructions. All the infection groups were analyzed in triplicates in each experiment, and total two independent experiments were conducted.

### T Cell Proliferation Assay

Two mice were injected intravenously with the p24 protein (30 μg/mouse). After 7 days, the splenocytes were washed with ice-cold FACS buffer [PBS containing 1% bovine serum albumin (BSA) and 1 mM EDTA] and blocked on ice for 30 min with a super block solution containing 10% rat sera (Sigma Aldrich), 10% goat sera (Gibco Invitrogen), 10% mouse sera (Sigma Aldrich), and 2.4G2 monoclonal antibody (10 µg/ml; BD Biosciences, San Diego, CA, USA). The cells were subsequently stained with BV421-conjugated anti-CD4 (Clone GK1.5, BD Biosciences) and PE-conjugated anti-CD8a (Clone 53-6.7, eBioscience, San Diego, CA, USA) for 30 min at 4°C and washed three times with ice-cold FACS buffer. The FACS AriaIII instrument (BD Biosciences) was used to sort the CD4 and CD8 T cell populations. One day before co-cultivation, immature BMDCs were also infected with the wild type, two rBCGs (i.e., rBCG-pMyong2-p24 and -pAL-p24) or rSmeg-pMyong2-p24 strains at an M.O.I. of 10 for 24 h. Proliferation assays were conducted using the fluorescent cytoplasmic tracking dye CFSE (Invitrogen, Carlsbad, CA, USA) as previously described (47). The sorted CD4 and CD8 T cells were stained with 5 µM CFSE for 4 min at 37°C and 4 min on ice. And then, the CFSE-labeled T cells and infected BMDCs were co-cultured for 4 days. Four days after co-cultivation of T cells and infected BMDCs, the co-cultured cells (in triplicate well) were blocked on ice for 30 min with a super block solution and stained with CD4 BV421-conjugated anti-CD4 (Clone GK1.5, BD Biosciences) and PE-conjugated anti-CD8a (Clone 53-6.7, eBioscience) for 30 min at 4°C. The cell cycle profiles were determined using FACS LSRFortessa (BD Biosciences) and analyzed using Flowjo software (Figure 3A). All the experiments were conducted in triplicate.

### IL-2 ELISA

The amounts of murine IL-2 in the co-cultured supernatants (in triplicate well) from the above T cell proliferation assay were also determined using ELISA according to the manufacturer’s instructions (BioLegend, USA). All the experiments were conducted in duplicate.

### Enzyme-Linked Immuno Spot (ELISPOT) Assay

Splenocytes from immunized mice (five mice/group) with wild type and rBCG strains were used to conduct an ELISPOT assay as previously described (63). In brief, 96-well ELISPOT plates (PVDF membrane) were coated with mouse IFN-γ (3 µg/ml, clone: AN-18) capture antibody (BD-Biosciences, San Diego, CA, USA) in PBS and incubated overnight at 4°C. The capture antibody was discarded, and the plates were washed with PBS containing 0.05% Tween-20 (PBST) and PBS (3 times each), and the plates were blocked with 200 µl of RPMI 1640 medium with 10% FBS for 3 h at 37°C. After blocking, 5 × 10^5^ cells of splenocytes from vaccinated mice were loaded into each well. For each treatment group, the cells were stimulated in triplicate with 5 µg/ml of purified p24 antigen or medium alone in a total volume of 200 µl. The plate was incubated at 37°C for 24 h. The cells were stimulated with 5 ng/ml of phorbol 12-myristate 13-acetate (PMA) (Sigma-Aldrich, St. Louis, MO, USA) and 500 ng/ml of ionomycin (Sigma-Aldrich) as a positive control. After washing with PBST and PBS (three times each), each well was treated with the biotin-labeled mouse IFN-γ (3 µg/ml, clone: XMG1.2) detection antibody (BD-Biosciences) and the plate was incubated overnight at 4°C. The wells were washed again and horseradish peroxidase (HRP)-conjugated streptavidin was added to each well. The HRP reaction was developed using the 3-amino-9-ethylcarbazole substrate reagent set (BD-Biosciences). The number of SFUs per well was automatically counted using an ELISPOT reader (AID ELISPOT reader, Strasburg, Germany). All the groups were analyzed in triplicates and two independent experiments were conducted.

### Determination of Cytokine Production in Mice Immunized With the rBCG Strains

The splenocytes from the immunized mice (five mice/group) were adjusted to a concentration of 1 × 10^6^ cells/well (96-well microplate, 200 µl volume, in triplicate) in RPMI 1640 medium with 10% FBS, and purified p24 protein was added at a concentration of 5 µg/ml for the *in vitro* stimulation. The cells were cultured, and the supernatants were harvested for the IL-2 (BioLegend, San Diego, CA, USA), TNF-α (eBioscience, San Diego, CA, USA), IL-6 (eBioscience), IL-10 (eBioscience), and IFN-γ (BioLegend) cytokine determination using ELISA kits. All the groups were analyzed in triplicates and two independent experiments were conducted.

### Serum Antibody Detection

To detect the serum antibody ratio, serum samples were collected from the immunized mice (five mice/group) using the heart puncture method after euthanasia *via* hyperventilation of CO_2_. The 96-well plate was coated overnight at 4°C with purified p24 protein (5 µg/ml) in 0.05 M carbonate-bicarbonate buffer (pH 9.6). The plate was washed three times with PBST and PBS and blocked at room temperature (RT) for 1 h with 5% BSA in PBST. The serum samples were diluted to a ratio of 1:10 in PBS and 100 µl were added to each well (in triplicate). The plate was incubated for 2 h at RT, washed three times with PBST and PBS, and incubated for 1 h with biotinylated rat anti-mouse IgG2a, IgG1 (BD Biosciences, 1:1,000 dilution), and total IgG (eBioscience, 1:1,000 dilution) antibodies. Then, the plate was washed again, incubated with HRP conjugated streptavidin (eBioscience) for 30 min at RT. After the final washing step, all the wells were reacted with the BD OptEIA substrate (BD Biosciences) for 10 min before the reaction was stopped using 1 N H_2_SO_4_. The OD was determined using a spectrometer at a wavelength of 450 nm (64).

### Cytotoxic T Lymphocyte (CTL) Assay

The induced CTL responses were determined as previously described (65) with slight modifications. In brief, for the effector cells, the splenocytes from the mice in each immunized group were pulsed using the major histocompatibility complex class I-restricted p24 peptide A9I (AMQMLKETI) (10 µg/ml; Peptron, Daejeon, South Korea) (52) and incubated for 6 days with IL-2 (30 U/ml; PeproTech, Rocky Hill, NJ, USA) at 37°C in a 5% CO_2_ incubator. The target cells, i.e., P815 cells (H-2^d^), were prepared by an incubation with the A9I peptide (10 µg/ml) for 2 h before the co-culture of the effector and target cells. Cell cytotoxicity was evaluated using an LDH assay in U bottom 96-well plates according to the manufacturer’s protocol (CytoTox 96 Non-Radioactive Cytotoxicity Assay; Promega, Madison, WI, USA). In brief, the effector cells (splenocytes stimulated by antigens) were added to the target cells (p24 pulsed P815 cells) in triplicate at different effector/target (E/T) ratios (ranging from 10:1, 20:1 to 50:1) for 6 h; then, the values of the LDH released from the cultured supernatants were detected using a spectrometer at 490 nm. The percentage of specific cell lysis was calculated using the following formula: [(Experimental − Effector spontaneous − Target spontaneous)/(Target maximum − Target spontaneous)] × 100 (%). All the groups were analyzed in triplicates and two independent experiments were conducted.

### Statistical Analysis

All presented data are expressed as the mean ± SD. Student’s *t*-test was used to compare the variance using Microsoft Excel software, and the differences were considered statistically significant at probability values less than 0.05.

## Ethics Statement

All animal experiments were carried out in accordance with the recommendations of institutional guidelines and the protocol approved by the Institutional Animal Care and Use Committee (IACUC; approval No. of SNU-160307-1-2) of the Institute of Laboratory Animal Resources at Seoul National University.

## Author Contributions

Byoung-JK and B-RK performed the experiments. Bum-JK and Y-HK designed and interpreted the experiments. Bum-JK wrote the manuscript.

## Conflict of Interest Statement

The authors declare that the research was conducted in the absence of any commercial or financial relationships that could be construed as a potential conflict of interest.

## References

[1] Chen LF Hoy J Lewin SR. Ten years of highly active antiretroviral therapy for HIV infection. Med J Aust (2007) 186(3):146–51. 17309405 10.5694/j.1326-5377.2007.tb00839.x

[2] Markowitz M Saag M Powderly WG Hurley AM Hsu A Valdes JM A preliminary study of ritonavir, an inhibitor of HIV-1 protease, to treat HIV-1 infection. N Engl J Med (1995) 333(23):1534–9. 10.1056/NEJM199512073332204 7477168

[3] Gamble LJ Matthews QL. Current progress in the development of a prophylactic vaccine for HIV-1. Drug Des Devel Ther (2010) 5:9–26. 10.2147/DDDT.S6959 21267356 PMC3023272

[4] Gray GE Laher F Lazarus E Ensoli B Corey L. Approaches to preventative and therapeutic HIV vaccines. Curr Opin Virol (2016) 17:104–9. 10.1016/j.coviro.2016.02.010 26985884 PMC5020417

[5] Kalams SA Buchbinder SP Rosenberg ES Billingsley JM Colbert DS Jones NG Association between virus-specific cytotoxic T-lymphocyte and helper responses in human immunodeficiency virus type 1 infection. J Virol (1999) 73(8):6715–20. 10400769 10.1128/jvi.73.8.6715-6720.1999 PMC112756

[6] Koup RA Safrit JT Cao Y Andrews CA McLeod G Borkowsky W Temporal association of cellular immune responses with the initial control of viremia in primary human immunodeficiency virus type 1 syndrome. J Virol (1994) 68(7):4650–5. 8207839 10.1128/jvi.68.7.4650-4655.1994 PMC236393

[7] Betts MR Nason MC West SM De Rosa SC Migueles SA Abraham J HIV nonprogressors preferentially maintain highly functional HIV-specific CD8+ T cells. Blood (2006) 107(12):4781–9. 10.1182/blood-2005-12-4818 16467198 PMC1895811

[8] Letvin NL Strategies for an HIV vaccine. J Clin Invest (2002) 110(1):15–20. 10.1172/JCI0215985 12093882 PMC151036

[9] Kutzler MA Weiner DB. DNA vaccines: ready for prime time? Nat Rev Genet (2008) 9(10):776–88. 10.1038/nrg2432 18781156 PMC4317294

[10] Nascimento IP Leite LC. Recombinant vaccines and the development of new vaccine strategies. Braz J Med Biol Res (2012) 45(12):1102–11. 10.1590/S0100-879X2012007500142 22948379 PMC3854212

[11] Medeiros MA Dellagostin OA Armoa GR Degrave WM De Mendonca-Lima L Lopes MQ Comparative evaluation of *Mycobacterium vaccae* as a surrogate cloning host for use in the study of mycobacterial genetics. Microbiology (2002) 148(Pt 7):1999–2009. 10.1099/00221287-148-7-1999 12101288

[12] Michelon A Conceicao FR Binsfeld PC da Cunha CW Moreira AN Argondizzo AP Immunogenicity of *Mycobacterium bovis* BCG expressing *Anaplasma marginale* MSP1a antigen. Vaccine (2006) 24(37–39):6332–9. 10.1016/j.vaccine.2006.05.028 16781025

[13] Daniel TM The history of tuberculosis. Respir Med (2006) 100(11):1862–70. 10.1016/j.rmed.2006.08.006 16949809

[14] Walker KB Brennan MJ Ho MM Eskola J Thiry G Sadoff J The second Geneva consensus: recommendations for novel live TB vaccines. Vaccine (2010) 28(11):2259–70. 10.1016/j.vaccine.2009.12.083 20074686

[15] Andersen P Kaufmann SH Novel vaccination strategies against tuberculosis. Cold Spring Harb Perspect Med (2014) 4(6):1–19. 10.1101/cshperspect.a018523 PMC4031959 24890836

[16] Eurosurveillance Editorial Team. WHO publishes global tuberculosis report 2013. Euro Surveill (2013) 18(43):20615. 24176622

[17] Connell ND Medina-Acosta E McMaster WR Bloom BR Russell DG Effective immunization against cutaneous leishmaniasis with recombinant bacille Calmette-Guerin expressing the *Leishmania* surface proteinase gp63. Proc Natl Acad Sci U S A (1993) 90(24):11473–7. 10.1073/pnas.90.24.11473 8265576 PMC48006

[18] Fennelly GJ Flynn JL ter Meulen V Liebert UG Bloom BR Recombinant bacille Calmette-Guerin priming against measles. J Infect Dis (1995) 172(3):698–705. 10.1093/infdis/172.3.698 7658061

[19] Langermann S Palaszynski SR Burlein JE Koenig S Hanson MS Briles DE Protective humoral response against pneumococcal infection in mice elicited by recombinant bacille Calmette-Guerin vaccines expressing pneumococcal surface protein A. J Exp Med (1994) 180(6):2277–86. 10.1084/jem.180.6.2277 7964500 PMC2191795

[20] Matsumoto S Yukitake H Kanbara H Yamada T Recombinant *Mycobacterium bovis Bacillus* Calmette-Guerin secreting merozoite surface protein 1 (MSP1) induces protection against rodent malaria parasite infection depending on MSP1-stimulated interferon gamma and parasite-specific antibodies. J Exp Med (1998) 188(5):845–54. 10.1084/jem.188.5.845 9730886 PMC2213399

[21] Nascimento IP Dias WO Mazzantini RP Miyaji EN Gamberini M Quintilio W Recombinant *Mycobacterium bovis* BCG expressing pertussis toxin subunit S1 induces protection against an intracerebral challenge with live *Bordetella pertussis* in mice. Infect Immun (2000) 68(9):4877–83. 10.1128/IAI.68.9.4877-4883.2000 10948100 PMC101688

[22] Stover CK Bansal GP Hanson MS Burlein JE Palaszynski SR Young JF Protective immunity elicited by recombinant bacille Calmette-Guerin (Bcg) expressing outer surface protein-A (Ospa) lipoprotein – a candidate Lyme-disease vaccine. J Exp Med (1993) 178(1):197–209. 10.1084/jem.178.1.197 8315378 PMC2191093

[23] Honda M Matsuo K Nakasone T Okamoto Y Yoshizaki H Kitamura K Protective immune-responses induced by secretion of a chimeric soluble-protein from a recombinant *Mycobacterium bovis Bacillus*-Calmette-Guerin vector candidate vaccine for human-immunodeficiency-virus type-1 in small animals. Proc Natl Acad Sci U S A (1995) 92(23):10693–7. 10.1073/pnas.92.23.10693 7479867 PMC40678

[24] Someya K Cecilia D Ami Y Nakasone T Matsuo K Burda S Vaccination of rhesus macaques with recombinant *Mycobacterium bovis Bacillus* Calmette-Guerin Env V3 elicits neutralizing antibody-mediated protection against simian-human immunodeficiency virus with a homologous but not a heterologous V3 motif. J Virol (2005) 79(3):1452–62. 10.1128/JVI.79.3.1452-1462.2005 15650171 PMC544111

[25] Daudel D Weidinger G Spreng S. Use of attenuated bacteria as delivery vectors for DNA vaccines. Expert Rev Vaccines (2007) 6(1):97–110. 10.1586/14760584.6.1.97 17280482

[26] Gupta UD Katoch VM McMurray DN. Current status of TB vaccines. Vaccine (2007) 25(19):3742–51. 10.1016/j.vaccine.2007.01.112 17321015

[27] Aagaard C Dietrich J Doherty M Andersen P. TB vaccines: current status and future perspectives. Immunol Cell Biol (2009) 87(4):279–86. 10.1038/icb.2009.14 19350048

[28] Chapman R Chege G Shephard E Stutz H Williamson AL. Recombinant *Mycobacterium bovis* BCG as an HIV vaccine vector. Curr HIV Res (2010) 8(4):282–98. 10.2174/157016210791208686 20353397 PMC3188323

[29] Dennehy M Williamson AL. Factors influencing the immune response to foreign antigen expressed in recombinant BCG vaccines. Vaccine (2005) 23(10):1209–24. 10.1016/j.vaccine.2004.08.039 15652663

[30] Cheynier R Gratton S Halloran M Stahmer I Letvin NL Wain-Hobson S. Antigenic stimulation by BCG vaccine as an in vivo driving force for SIV replication and dissemination. Nat Med (1998) 4(4):421–7. 10.1038/Nm0498-421 9546787

[31] Zhou DJ Shen Y Chalifoux L Lee-Parritz D Simon M Sehgal PK *Mycobacterium bovis* bacille Calmette-Guerin enhances pathogenicity of simian immunodeficiency virus infection and accelerates progression to AIDS in macaques: a role of persistent T cell activation in AIDS pathogenesis. J Immunol (1999) 162(4):2204–16. 9973496

[32] Kim BJ Math RK Jeon CO Yu HK Park YG Kook YH *Mycobacterium yongonense* sp. nov., a slow-growing non-chromogenic species closely related to *Mycobacterium intracellulare*. Int J Syst Evol Microbiol (2013) 63(Pt 1):192–9. 10.1099/ijs.0.037465-0 22427442

[33] Kim BJ Kim BR Lee SY Kim GN Kook YH Kim BJ. Molecular taxonomic evidence for two distinct genotypes of *Mycobacterium yongonense* via genome-based phylogenetic analysis. PLoS One (2016) 11(3):e0152703. 10.1371/journal.pone.0152703 27031100 PMC4816341

[34] Kim BJ Kim K Kim BR Kook YH Kim BJ. Identification of ISMyo2, a novel insertion sequence element of IS21 family and its diagnostic potential for detection of *Mycobacterium yongonense*. BMC Genomics (2015) 16:794. 10.1186/s12864-015-1978-2 26472562 PMC4608216

[35] Kim BJ Kim BR Lee SY Seok SH Kook YH Kim BJ. Whole-genome sequence of a novel species, *Mycobacterium yongonense* DSM 45126^T^. Genome Announc (2013) 1(4):e00604–13. 10.1128/genomeA.00604-13 23929490 PMC3738906

[36] Kim BJ Hong SH Kook YH Kim BJ. Molecular evidence of lateral gene transfer in *rpoB* gene of *Mycobacterium yongonense* strains via multilocus sequence analysis. PLoS One (2013) 8(1):e51846. 10.1371/journal.pone.0051846 23382812 PMC3561371

[37] Kim BJ Kim BR Kook YH Kim BJ. Role of the DNA mismatch repair gene *MutS4* in driving the evolution of *Mycobacterium yongonense* type I via homologous recombination. Front Microbiol (2017) 8:2578. 10.3389/Fmicb.2017.02578 29326683 PMC5742357

[38] Rauzier J Monizpereira J Gicquelsanzey B Complete nucleotide-sequence of Pal5000, a plasmid from *Mycobacterium fortuitum*. Gene (1988) 71(2):315–21. 10.1016/0378-1119(88)90048-0 3224826

[39] Lee H Kim BJ Kim BR Kook YH Kim BJ The development of a novel *Mycobacterium-Escherichia coli* shuttle vector system using pMyong2, a linear plasmid from *Mycobacterium yongonense* DSM 45126(T). PLoS One (2015) 10(3):e0122897 10.1371/journal.pone.0122897 25822634 PMC4378964

[40] Kim BJ Gong JR Kim GN Kim BR Lee SY Kook YH Recombinant *Mycobacterium smegmatis* with a pMyong2 vector expressing human immunodeficiency virus type I Gag can induce enhanced virus-specific immune responses. Sci Rep (2017) 7:44776. 10.1038/Srep44776 28300196 PMC5353558

[41] Kawahara M Matsuo K Nakasone T Hiroi T Kiyono H Matsumoto S Combined intrarectal/intradermal inoculation of recombinant *Mycobacterium bovis Bacillus* Calmette-Guerin (BCG) induces enhanced immune responses against the inserted HIV-1 V3 antigen. Vaccine (2002) 21(3–4):158–66. 10.1016/S0264-410X(02)00465-6 12450689

[42] Salk J Bretscher PA Salk PL Clerici M Shearer GM A strategy for prophylactic vaccination against HIV. Science (1993) 260(5112):1270–2. 10.1126/science.8098553 8098553

[43] Edwards BH Bansal A Sabbaj S Bakari J Mulligan MJ Goepfert PA. Magnitude of functional CD8+ T-cell responses to the Gag protein of human immunodeficiency virus type 1 correlates inversely with viral load in plasma. J Virol (2002) 76(5):2298–305. 10.1128/jvi.76.5.2298-2305.2002 11836408 PMC135950

[44] Kiepiela P Ngumbela K Thobakgale C Ramduth D Honeyborne I Moodley E CD8(+) T-cell responses to different HIV proteins have discordant associations with viral load. Nat Med (2007) 13(1):46–53. 10.1038/nm1520 17173051

[45] Zuniga R Lucchetti A Galvan P Sanchez S Sanchez C Hernandez A Relative dominance of Gag p24-specific cytotoxic T lymphocytes is associated with human immunodeficiency virus control. J Virol (2006) 80(6):3122–5. 10.1128/Jvi.80.6.3122-3125.2006 16501126 PMC1395458

[46] Andreu N Zelmer A Fletcher T Elkington PT Ward TH Ripoll J Optimisation of bioluminescent reporters for use with mycobacteria. PLoS One (2010) 5(5):e10777. 10.1371/journal.pone.0010777 20520722 PMC2875389

[47] Quah BJ Warren HS Parish CR. Monitoring lymphocyte proliferation in vitro and in vivo with the intracellular fluorescent dye carboxyfluorescein diacetate succinimidyl ester. Nat Protoc (2007) 2(9):2049–56. 10.1038/nprot.2007.296 17853860

[48] Mountford AP Fisher A Wilson RA. The profile of IgG1 and IgG2a antibody responses in mice exposed to *Schistosoma mansoni*. Parasite Immunol (1994) 16(10):521–7. 10.1111/j.1365-3024.1994.tb00306.x 7870462

[49] Finkelman FD Holmes J Katona IM Urban JF Jr Beckmann MP Park LS Lymphokine control of in vivo immunoglobulin isotype selection. Annu Rev Immunol (1990) 8:303–33. 10.1146/annurev.iy.08.040190.001511 1693082

[50] Germann T Bongartz M Dlugonska H Hess H Schmitt E Kolbe L Interleukin-12 profoundly up-regulates the synthesis of antigen-specific complement-fixing IgG2a, IgG2b and IgG3 antibody subclasses in vivo. Eur J Immunol (1995) 25(3):823–9. 10.1002/eji.1830250329 7705414

[51] Matsuo K Yasutomi Y *Mycobacterium bovis* bacille Calmette-Guerin as a vaccine vector for global infectious disease control. Tuberc Res Treat (2011) 2011:574591 10.1155/2011/574591 22567267 PMC3335490

[52] Kanekiyo M Matsuo K Hamatake M Hamano T Ohsu T Matsumoto S Mycobacterial codon optimization enhances antigen expression and virus-specific immune responses in recombinant *Mycobacterium bovis* bacille Calmette-Guerin expressing human immunodeficiency virus type 1 Gag. J Virol (2005) 79(14):8716–23. 10.1128/JVI.79.14.8716-8723.2005 15994765 PMC1168777

[53] Cespedes PF Rey-Jurado E Espinoza JA Rivera CA Canedo-Marroquin G Bueno SM A single, low dose of a cGMP recombinant BCG vaccine elicits protective T cell immunity against the human respiratory syncytial virus infection and prevents lung pathology in mice. Vaccine (2017) 35(5):757–66. 10.1016/j.vaccine.2016.12.048 28065474

[54] Power CA Wei GJ Bretscher PA. Mycobacterial dose defines the Th1/Th2 nature of the immune response independently of whether immunization is administered by the intravenous, subcutaneous, or intradermal route. Infect Immun (1998) 66(12):5743–50. 9826349 10.1128/iai.66.12.5743-5750.1998 PMC108725

[55] Bastos RG Dellagostin OA Barletta RG Doster AR Nelson E Osorio FA. Construction and immunogenicity of recombinant *Mycobacterium bovis* BCG expressing GP5 and M protein of porcine reproductive respiratory syndrome virus. Vaccine (2002) 21(1–2):21–9. 10.1016/S0264-410x(02)00443-7 12443659

[56] Klein SL Marriott I Fish EN. Sex-based differences in immune function and responses to vaccination. Trans R Soc Trop Med Hyg (2015) 109(1):9–15. 10.1093/trstmh/tru167 25573105 PMC4447843

[57] Fish EN. The X-files in immunity: sex-based differences predispose immune responses. Nat Rev Immunol (2008) 8(9):737–44. 10.1038/nri2394 18728636 PMC7097214

[58] Snapper SB Melton RE Mustafa S Kieser T Jacobs WR Jr. Isolation and characterization of efficient plasmid transformation mutants of *Mycobacterium smegmatis*. Mol Microbiol (1990) 4(11):1911–9. 10.1111/j.1365-2958.1990.tb02040.x 2082148

[59] Aghababa H Mobarez AM Behmanesh M Khoramabadi N Mobarhan M. Production and purification of mycolyl transferase B of *Mycobacterium tuberculosis*. Tanaffos (2011) 10(4):23–30. 25191384 PMC4153168

[60] Kurien BT Scofield RH. Heat mediated quick Coomassie blue protein staining and destaining of SDS-PAGE gels. Indian J Biochem Biophys (1998) 35(6):385–9. 10412235

[61] Madaan A Verma R Singh AT Jain SK Jaggi M A stepwise procedure for isolation of murine bone marrow and generation of dendritic cells. J Biol Methods (2014) 1(1):1–6. 10.14440/jbm.2014.12

[62] Kuroishi A Saito A Shingai Y Shioda T Nomaguchi M Adachi A Modification of a loop sequence between alpha-helices 6 and 7 of virus capsid (CA) protein in a human immunodeficiency virus type 1 (HIV-1) derivative that has simian immunodeficiency virus (SIVmac239) vif and CA alpha-helices 4 and 5 loop improves replication in cynomolgus monkey cells. Retrovirology (2009) 6:70. 10.1186/1742-4690-6-70 19650891 PMC2731049

[63] Power CA Grand CL Ismail N Peters NC Yurkowski DP Bretscher PA. A valid ELISPOT assay for enumeration of ex vivo, antigen-specific, IFNgamma-producing T cells. J Immunol Methods (1999) 227(1–2):99–107. 10.1016/S0022-1759(99)00074-5 10485258

[64] Deng Y Bao L Yang X. Evaluation of immunogenicity and protective efficacy against *Mycobacterium tuberculosis* infection elicited by recombinant *Mycobacterium bovis* BCG expressing human interleukin-12p70 and early secretory antigen target-6 fusion protein. Microbiol Immunol (2011) 55(11):798–808. 10.1111/j.1348-0421.2011.00376.x 21831202

[65] Fan XL Yu TH Gao Q Yao W Immunological properties of recombinant *Mycobacterium bovis Bacillus* Calmette-Guerin strain expressing fusion protein IL-2-ESAT-6. Acta Biochim Biophys Sin (Shanghai) (2006) 38(10):683–90. 10.1111/j.1745-7270.2006.00217.x 17033714

