# Supplementary material for: Development of a Live Recombinant BCG Expressing Human Immunodeficiency Virus Type 1 (HIV-1) Gag Using a pMyong2 Vector System: Potential Use As a Novel HIV-1 Vaccine
Source: Front Immunol. 2018 Mar 27;9:643. doi: 10.3389/fimmu.2018.00643 (PMC5880907; doi:10.3389/fimmu.2018.00643)
Supplement: Supplementary file 1 [file presentation_1.PDF]

Supplementary Figures

Potential of a recombinant BCG using a pMyong2 vector system expressing HIV-1 Gag in vaccine application for HIV-1 infection

Byoung-Jun Kim, Bo-Ram Kim, Yoon-Hoh Kook and Bum-Joon Kim*

Department of Microbiology and Immunology, Biomedical Sciences, Liver Research Institute and Cancer Research Institute, College of Medicine, Seoul National University, Seoul, Korea

***Author for correspondence:** Bum-Joon Kim, phD,

E-mail : kbumjoon@snu.ac.kr.

Supplementary Table S1. Cytokine levels, as determined by ELISA, in vitro stimulated splenocytes with p24 from each immunized group of mice (five mice/group). Data are representative of two independent experiments. Means ± SD are shown.

Groups	IL-2 (pg/ml)		IFN-γ (pg/ml)		TNF-α (pg/ml)		IL-6 (pg/ml)		IL-10 (pg/ml)	
	Day 1	Day 3	Day 1	Day 3	Day 1	Day 3	Day 1	Day 3	Day 1	Day 3
No treat	2.67 ± 0.09	1.29 ± 0.37	10.02 ± 1.41	11.32 ± 0.42	35.49 ± 0.87	57.45 ± 6.09	10.10 ± 1.16	9.92 ± 0.25	55.68 ± 7.40	60.27 ± 0.91
BCG	5.42 ± 0.24	9.42 ± 1.44	12.55 ± 0.44	10.95 ± 0.52	51.85 ± 8.62	96.32 ± 12.79	32.98 ± 1.91	33.22 ± 0.52	104.98 ± 24.04	134.77 ± 6.17
rBCG-pAL-p24	15.90 ± 0.06	29.53 ± 4.90	31.31 ± 1.12	29.75 ± 1.72	142.77 ± 13.00	207.43 ± 15.63	111.08 ± 22.47	107.28 ± 11.02	135.39 ± 13.73	145.24 ± 12.81
rBCG-pMyong2-p24	18.70 ± 3.44	33.86 ± 1.38	35.38 ± 2.02	56.50 ± 1.42	160.31 ± 14.18	238.96 ± 5.58	148.41 ± 22.76	166.26 ± 23.74	140.26 ± 12.58	149.74 ± 9.40
rSmeg-pMyong2-p24	16.81 ± 0.41	29.84 ± 0.37	33.15 ± 1.80	46.70 ± 8.06	162.40 ± 13.17	242.90 ± 0.64	126.80 ± 3.76	155.76 ± 27.02	138.91 ± 17.38	151.66 ± 23.25

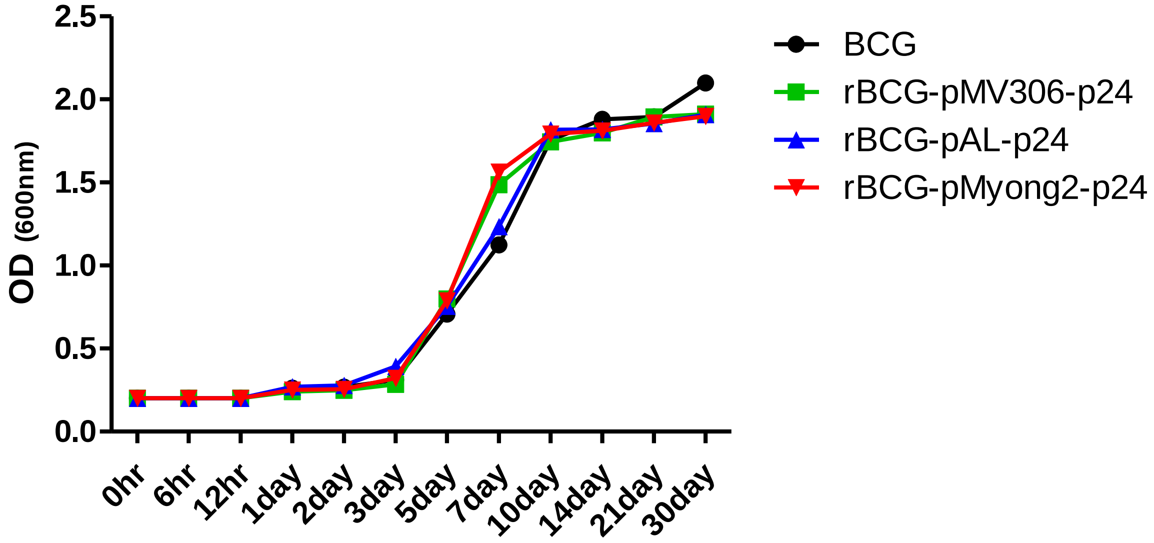

Supplementary Figure S1. The growth curve of p24 rBCG strains in 7H9 broth supplemented with ADC and 100 μg/ml of kanamycin. In the case of wild- type BCG culture, kanamycin was excluded from 7H9 broth. To establish the growth curve, culture aliquots were taken at each time point and the OD_{600} was measured.

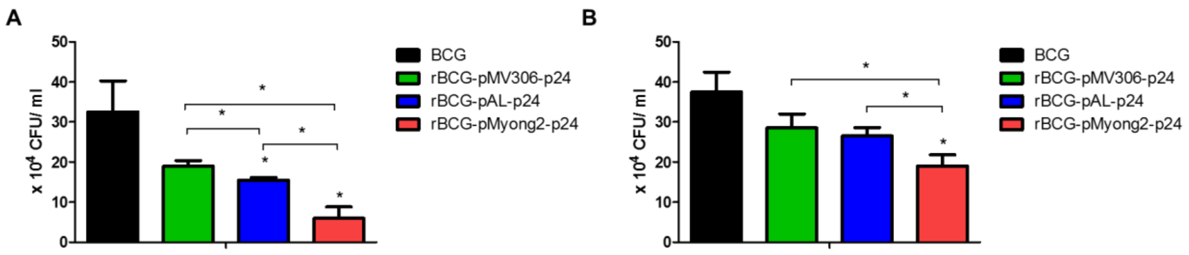

Supplementary Figure S2. Comparison of CFU levels of p24 rBCG strains in infection of (A) murine macrophage J774A.1 and (B) mouse bone-marrow derived dendritic cells. Data are representative of two independent experiments. Means ± SD are shown. *$P < 0.05$; **$P < 0.01$; ***$P < 0.001$ (Student's *t*-test).

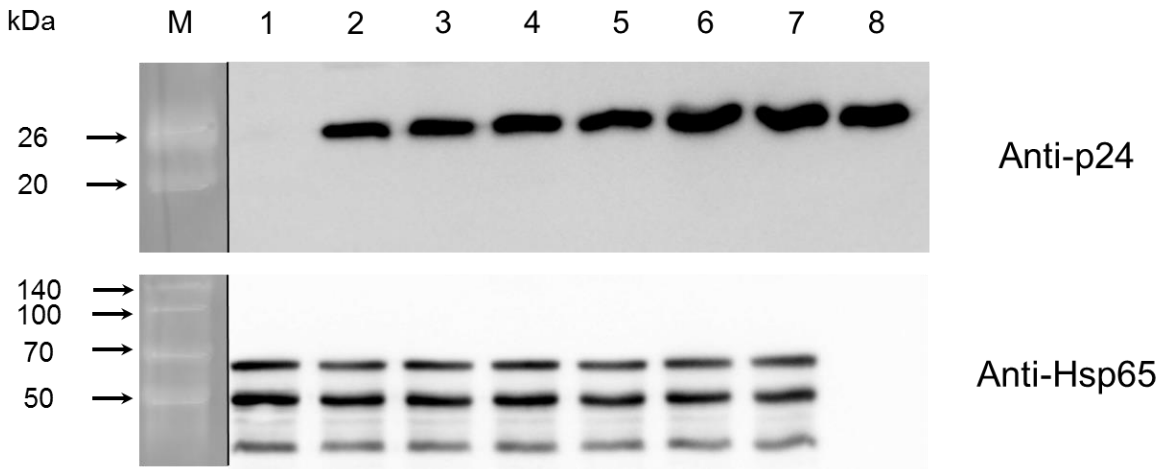

Supplementary Figure S3. Stability confirmation of p24 expression in rBCG-pMyong2-p24 strain passaged on 7H10 agar plate with kanamycin by western blot. Proteins were extracted from wild-type BCG (lane 1) and rBCG-pMyong2-p24 strain at each passage point (lane 2, first passage; lane 3, after 4th passages; lane 4, after 6th passages; lane 5, after 8th passages; lane 6, after 10th passages; lane 7, after 12th passages). Purified p24 protein was used as a positive control (lane 8). M, molecular weight standard (Elpis Bio, Taejeon, Korea; DokDo-MARKTM). The membrane was cropped and probed with Hsp65 antibody (Abcam) as an internal control at the upper size membrane. Distinct membranes were separated by white space. And, marker lane was separated by a vertical black line.

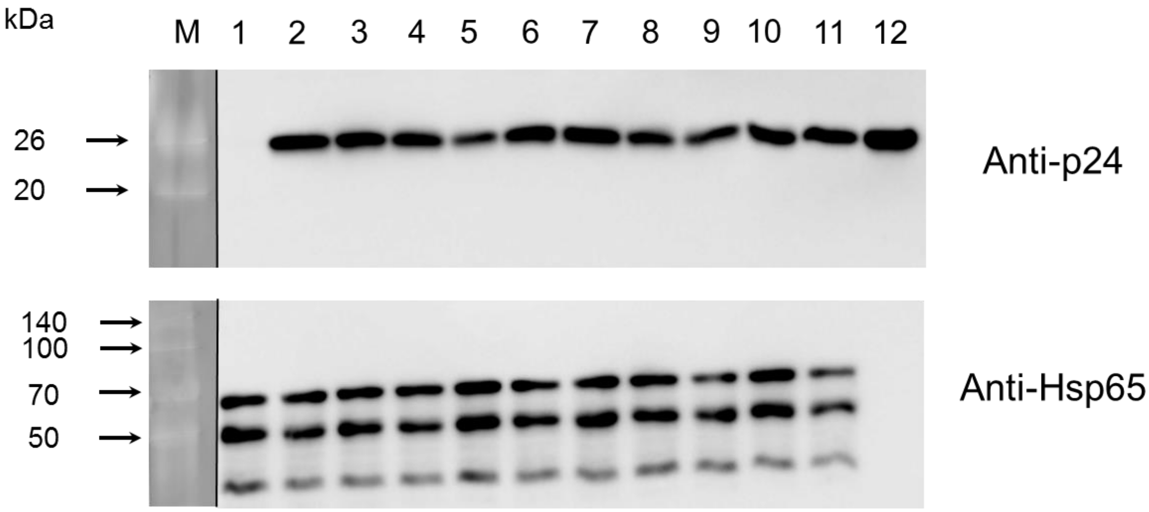

Supplementary Figure S4. Stability confirmation of p24 expression in rBCG-pMyong2-p24 strain passaged on 7H10 agar plate without kanamycin by Western blot. Proteins were extracted from wild-type BCG (lane 1) and rBCG-pMyong2-p24 strain at each passage point (lane 2, first passage; lane 3, after 4th passages; lane 4, after 5th passages; lane 5, after 6th passages; lane 6, after 7th passages; lane 7, after 8th passages; lane 8, after 9th passages; lane 9, after 10th passages; lane 10, after 11th passages; lane 11, after 12th passages). Purified p24 protein was used as a positive control (lane 12). M, molecular weight standard (Elpis Bio, Taejeon, Korea; DokDo-MARKTM). The membrane was cropped and probed with Hsp65 antibody (Abcam) as an internal control at the upper size membrane. Distinct membranes were separated by white space. And, marker lane was separated by a vertical black line.

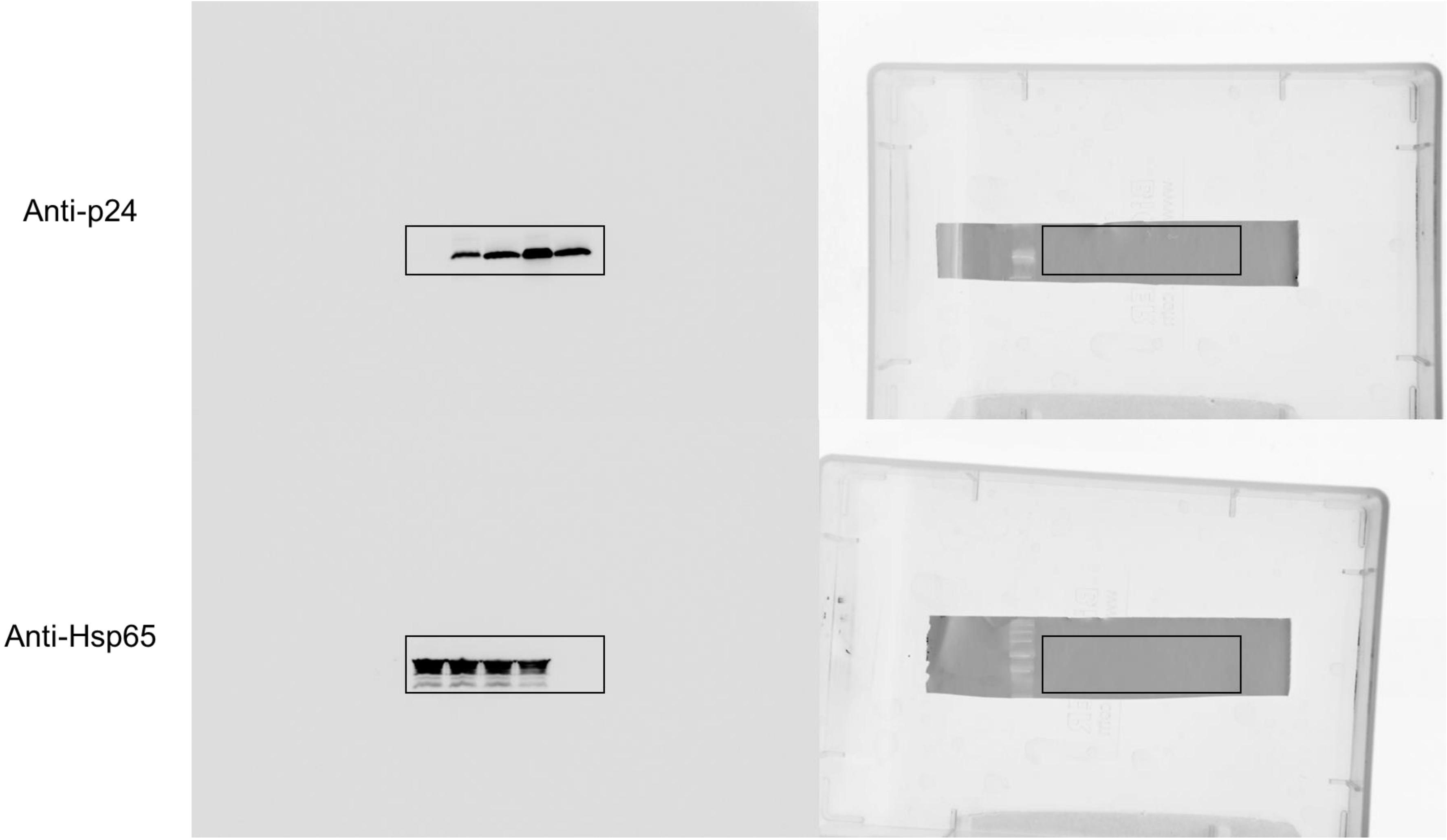

Supplementary Figure S5. Uncropped, full length original blots of the cropped image of Figure 2B presented in the manuscript. The blots were performed with the indicated antibodies. The cropped area was indicated by the solid lines.

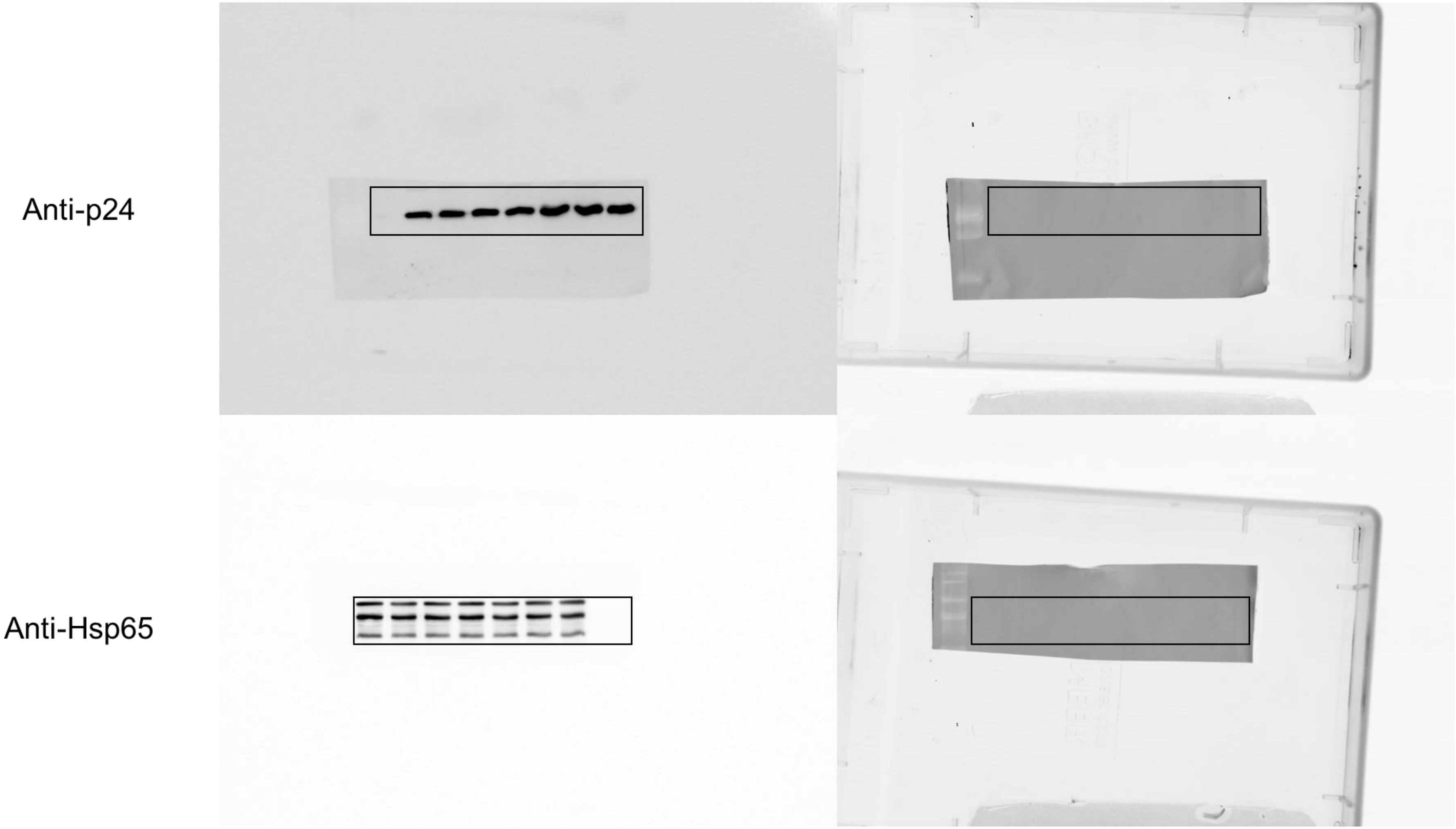

Supplementary Figure S6. Uncropped, full length original blots of the cropped image of Supplementary Figure S3 presented in the manuscript.

The blots were performed with the indicated antibodies. The cropped area was indicated by the solid lines.

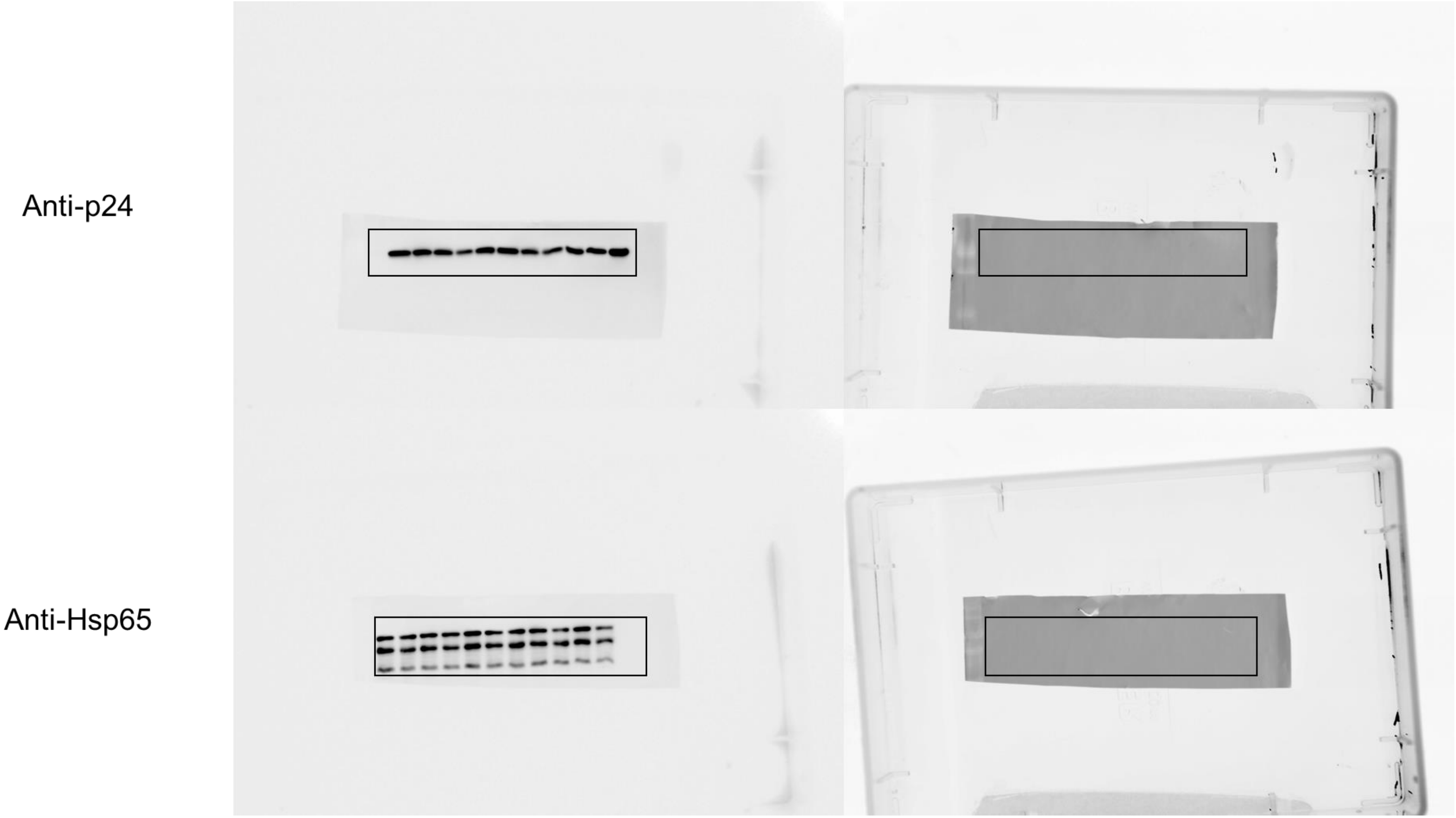

Supplementary Figure S7. Uncropped, full length original blots of the cropped image of Supplementary Figure S4 presented in the manuscript.

The blots were performed with the indicated antibodies. The cropped area was indicated by the solid lines.

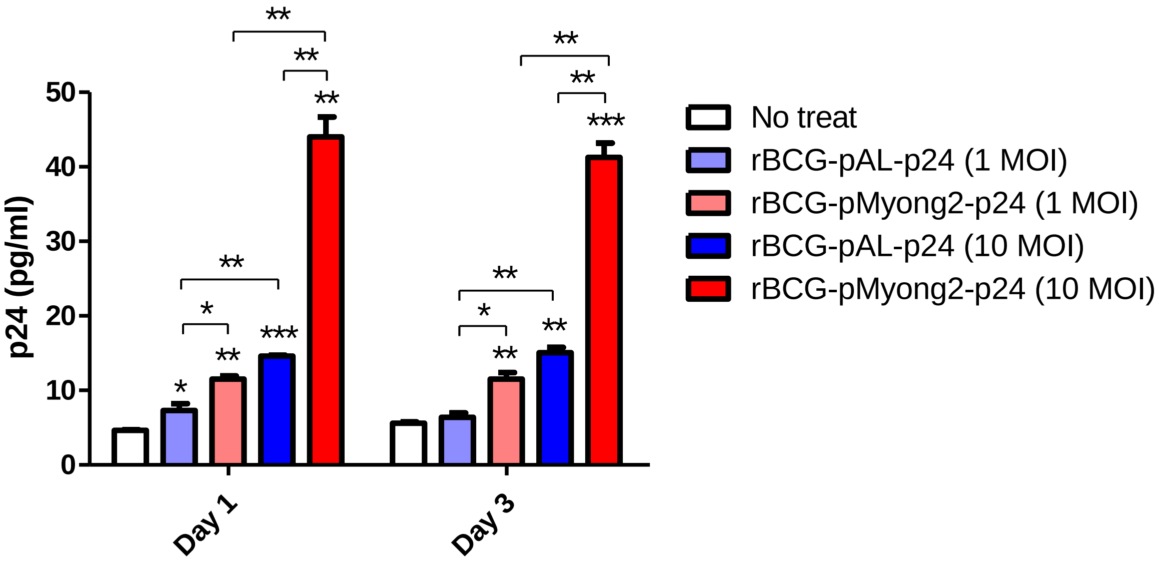

Supplementary Figure S8. Comparing the expression levels of p24 from BMDCs infected with different M.O.I. (1 and 10 M.O.I.) of rBCG-pAL-p24 and rBCG-pMyong2-p24 strains for 1 and 3 days using ELISA. Data are shown with Means ± SD in duplicate wells. *$P < 0.05$; **$P < 0.01$; ***$P < 0.001$ (Student's *t*-test).

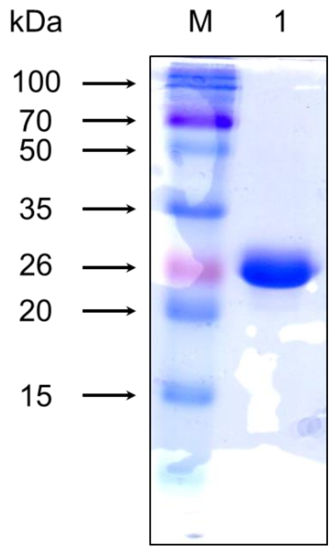

Supplementary Figure S9. Purity confirmation of p24 protein used in this study by SDS-PAGE and Coomassie brilliant blue staining. M, molecular weight standard (Elpis Bio, Taejeon, Korea; DokDo-MARK^{TM}). Lane 1, purified p24 protein (10 μg).
